# Digital Health Supportive Care Interventions for Breast Cancer Survivors: An Umbrella Review of Systematic Reviews and Meta-Analyses

**DOI:** 10.3390/cancers18213434

**Published:** 2026-10-23

**Authors:** Mattias Wei Ren Kon, Joan Ern Xin Tan, Charmaine Su Min Tan, Rachel Qiao Jie Lim, Kevin Xiang Zhou, Nan Liu, Qin Xiang Ng, Serene Si Ning Goh

**Affiliations:** 1 Yong Loo Lin School of Medicine, National University of Singapore, 10 Medical Drive, Singapore 117597, Singapore; mattias.kon@u.nus.edu (M.W.R.K.);; 2 Faculty of Medicine and Health Sciences, McGill University, Montreal, QC H3G 2M1, Canada; 3 Programme in Health Services and Systems Research, Duke-NUS Medical School, 8 College Road, Singapore 169857, Singapore; 4 Department of Biostatistics and Bioinformatics, Duke University, Durham, NC 27710, USA; 5 Research and Translational Innovation Office, SingHealth Community Hospitals, 10 Hospital Blvd, Singapore 168582, Singapore; 6 Saw Swee Hock School of Public Health, National University of Singapore, 12 Science Drive 2, Singapore 117549, Singapore; 7 Department of General Surgery, National University Hospital, 5 Lower Kent Ridge Road, Singapore 119074, Singapore

**Keywords:** breast cancer, cancer survivorship, digital health, mHealth, telehealth, wearable activity trackers, quality of life, physical activity, psychosocial outcomes, umbrella review

## Abstract

Background/Objectives: Digital health interventions are a common form of supportive care for breast cancer survivors and may improve quality of life (QoL), physical activity and psychosocial outcomes, yet the evidence is fragmented across many systematic reviews. This umbrella review synthesised systematic reviews and meta-analyses of digital health interventions for breast cancer survivors to characterise their modalities, effectiveness and readiness for real-world implementation. Methods: Following a prospectively registered protocol (PROSPERO CRD420251185022), six databases were searched from inception to July 2025 for systematic reviews or meta-analyses evaluating any digital health intervention in adult breast cancer survivors and reporting QoL, physical or psychosocial outcomes. Methodological quality was appraised with AMSTAR-2, certainty of evidence with GRADE, overlap of primary studies with the corrected covered area (CCA), and findings were synthesised narratively. Results: Twenty-three systematic reviews were included, encompassing 459 primary study occurrences (333 unique studies; CCA 1.7%, slight overlap) and 44,560 participants across the 15 reviews that reported sample sizes. Interventions comprised online programmes, mobile applications (mHealth), telephone-based interventions, wearable activity trackers, virtual reality and an artificial intelligence chatbot. Across the included reviews, digital health interventions generally demonstrated favourable effects on QoL, physical activity, and psychosocial outcomes, although findings varied across intervention modalities and individual outcomes. Methodological confidence was low in 19 of 23 reviews (83%), limiting the robustness of these findings. Engagement or adherence was reported by 83% of reviews, but economic data (13%), equity considerations (35%) and health-system integration (43%) were rarely addressed. Conclusions: Digital health interventions show promise as supportive care tools for breast cancer survivors, particularly for improving QoL, physical activity, and psychosocial well-being. However, methodological limitations and evidence gaps regarding long-term engagement and cost-effectiveness persist. Future work should evaluate their integration into survivorship pathways, report engagement as standard, and generate the implementation and economic evidence that integration requires.

## Simple Summary

Many women who complete breast cancer treatment continue to live with fatigue, pain, low mood, anxiety and fear of recurrence. Digital tools such as smartphone apps, websites, telephone coaching, wearable activity trackers and virtual reality programmes are increasingly used to help survivors manage these problems from home. Systematic reviews have examined these tools, but their findings have not been brought together across all types of tool. We searched six databases and summarised 23 systematic reviews covering 333 unique studies. Across every type of tool, survivors who used digital interventions reported better quality of life, more physical activity and less anxiety and depression than those receiving usual care alone. However, few reviews examined whether survivors kept using the tools over time, what they cost, or how they could be built into routine follow-up care. Future studies should test digital tools in the real-world settings in which they are meant to be used.

## 1. Introduction

Breast cancer is the most common cancer in women globally and causes substantial morbidity, mortality and economic strain on healthcare systems, with incidence and mortality rising most steeply in low- and middle-income countries (LMICs) [1]. Whilst research has traditionally focused on cancer therapeutics and treatment strategies, supportive care for survivors who have completed breast cancer treatment has attracted increasing attention.

Supportive care interventions target three broad categories of problems faced by breast cancer survivors: physical, psychological and social sequelae [2,3]. Physical concerns commonly include fatigue, pain, lymphoedema, premature menopause and vasomotor symptoms [4,5,6]. Psychological concerns include anxiety, depression and fear of recurrence, while social concerns include greater needs for home care, workplace support and financial support [7,8,9,10]. To address these concerns, a range of approaches has been studied, including psychological (e.g., cognitive behavioural therapy (CBT)) [11,12], physical (e.g., yoga and exercise) [13,14], educational [15], nutritional [16] and digital interventions.

Digital health interventions refer to the use of electronic and communication technologies to deliver healthcare information, monitoring, rehabilitation, and supportive care outside conventional face-to-face clinical encounters. With the rise of technology, they have been increasingly adopted as a preferred mode of delivering patient care across health systems [17]. They encompass several distinct modalities, such as mobile applications (mHealth), web-based platforms, telehealth, wearable devices and social media programmes, that provide supportive care by delivering health information, monitoring symptoms, facilitating communication or assisting in self-management [18,19]. For example, a survivor experiencing fatigue after chemotherapy may use a mobile application to record symptoms, receive activity recommendations, and track progress, while another may participate in telephone-based counselling to address anxiety or fear of recurrence [18,19,20]. Beyond breast cancer, digital health interventions have been widely adopted across the spectrum of oncological care, with reported benefits including better control of post-chemotherapy side effects, improved quality of life (QoL) and psychological reassurance, among other patient-reported outcomes [18,19].

The mechanisms through which digital health interventions deliver these benefits have been examined previously, including their ability to overcome barriers to access and their scalability and cost-effectiveness [20,21]. In a 2020 review, Penedo et al. [21] described how digital health tools eliminate geographic barriers [22,23], provide flexible asynchronous care [24,25], bridge the gap to primary care follow-up [26] and reach underserved communities and populations [27,28,29]. Moreover, some mHealth tools have shown remarkably low marginal costs per user alongside reduced hospitalisations and healthcare burden, demonstrating both efficacy and cost-effectiveness [30,31,32,33,34]. Digital health technologies therefore show promise in managing the financial toxicity of cancer care while offering high accessibility and ease of remote follow-up [35].

However, the wide range of interventions and outcome measures across the many published studies, together with review scopes that often extend beyond breast cancer survivors, has prevented a consistent evaluation of each mode of digital health intervention. An umbrella review, which synthesises and critically appraises existing systematic reviews and meta-analyses, offers a robust approach for consolidating this evidence, identifying consistent findings and highlighting gaps in the literature.

The objectives of this umbrella review were (1) to synthesise evidence from systematic reviews and meta-analyses of digital health interventions among breast cancer survivors; (2) to evaluate their effects on QoL and on physical and psychosocial outcomes; and (3) to identify implications for clinical practice, health policy and future research in breast cancer survivorship care.

## 2. Materials and Methods

### 2.1. Study Design and Registration

This umbrella review (an overview of systematic reviews and meta-analyses) was conducted in accordance with the guidance on overviews of reviews in the Cochrane Handbook for Systematic Reviews of Interventions and is reported according to the Preferred Reporting Items for Systematic Reviews and Meta-Analyses (PRISMA) 2020 statement [36,37]. The protocol was prospectively registered with the International Prospective Register of Systematic Reviews (PROSPERO; registration number CRD420251185022).

### 2.2. Eligibility Criteria

Systematic reviews or meta-analyses were included if they (1) explicitly described systematic methods for literature identification, selection and synthesis; (2) focused on adult (≥18 years) breast cancer survivors who had completed primary treatment (surgery, radiotherapy and/or chemotherapy); (3) evaluated digital health interventions of any type, including mHealth, telehealth, web-based, wearable, virtual reality (VR)-based or artificial intelligence (AI)-based interventions; (4) reported health-related outcomes such as QoL, physical health or psychosocial health; and (5) included randomised controlled trials (RCTs), prospective cohorts or other controlled comparative designs. Reviews that exclusively examined non-breast-cancer populations, non-interventional designs or non-digital supportive care were excluded unless breast-cancer-specific sub-analyses were reported. Only English-language, peer-reviewed publications were considered; grey literature and conference abstracts were excluded.

Although the eligibility criteria specified survivors who had completed primary treatment, the final review also included systematic reviews encompassing mixed treatment-phase populations, including individuals undergoing active treatment and those in post-treatment follow-up. This represents a broadening of the original population eligibility criterion to include evidence relevant to supportive care across the breast cancer continuum. The deviation is reported transparently, and the implications of including mixed treatment-phase populations are considered when interpreting the findings.

### 2.3. Search Strategy and Study Selection

Six databases (CINAHL, Cochrane Library, Embase, PsycINFO, PubMed/MEDLINE and Scopus) were searched from inception to 31 July 2025. Search terms combined controlled vocabulary and free-text terms for breast cancer, digital health and systematic reviews; the full search strategy for each database is provided in Table S2. All records were imported into Covidence (Veritas Health Innovation, Melbourne, Australia). After removal of duplicates, titles, abstracts and full texts were screened for eligibility by three independent investigators (M.W.R.K., J.E.X.T. and C.S.M.T.). Disagreements were resolved through discussion and adjudication by a senior investigator (Q.X.N. or S.S.N.G.).

### 2.4. Data Extraction

A standardised extraction template was used to capture review characteristics (first author, year, databases searched, number and design of primary studies, intervention type, comparators, study population, intervention duration, outcomes, key findings and quality-appraisal methods) together with implementation-relevant characteristics (theoretical basis and behaviour change techniques, intervention intensity, personalisation, automation and human support, engagement metrics, platform developer, integration with the healthcare system, digital literacy requirements, cost to users, scalability, maintenance, equity considerations, attrition, follow-up duration and economic data). Data from each included review were extracted independently by three investigators (M.W.R.K., J.E.X.T. and C.S.M.T.) and cross-checked by a senior investigator (S.S.N.G.). The extended extraction data are provided in Table S5. Interventions were categorised as online or web-based, mobile application (mHealth), telephone-based, wearable activity tracker, VR-based or AI chatbot interventions. The primary outcomes of interest were QoL, physical health parameters (physical activity, weight control, fatigue, aerobic fitness and anthropometry) and psychosocial health parameters (anxiety, depression, stress and mood).

### 2.5. Methodological Quality and Certainty of Evidence

Two investigators (M.W.R.K. and R.Q.J.L.) independently assessed the methodological quality of the included reviews using A MeaSurement Tool to Assess systematic Reviews, version 2 (AMSTAR-2), which covers protocol registration, search comprehensiveness, risk-of-bias assessment and appropriateness of synthesis, among other domains [38]. Disagreements were resolved by discussion until consensus was reached. Overall confidence in the results of each review was rated as critically low, low, moderate or high.

Certainty of evidence for the key outcomes of each review was rated by the same two investigators using the Grading of Recommendations, Assessment, Development and Evaluation (GRADE) approach [39], considering risk of bias, imprecision, indirectness, inconsistency and magnitude of effect, and taking into account the GRADE ratings reported by the original reviews where available. GRADEpro GDT software (McMaster University and Evidence Prime, Hamilton, ON, Canada) was used to support the appraisal. Certainty was rated as very low, low, moderate or high.

### 2.6. Overlap of Primary Studies

To account for duplication of primary studies across the included reviews, the degree of overlap in the evidence base was assessed. For each review, the list of included primary studies was extracted and a citation matrix was constructed with reviews as columns and primary studies as rows (Table S4). Primary studies were de-duplicated by author name, publication year, title, trial registration number (where available) and DOI. The corrected covered area (CCA) was then calculated as CCA = (N − r)/(rc − r), where N is the total number of included study occurrences across reviews, r is the number of unique primary studies and c is the number of included reviews. Overlap was interpreted as slight (CCA < 5%), moderate (5–10%), high (10–15%) or very high (>15%). Where substantial overlap was identified, findings were interpreted cautiously to avoid double-counting the same primary evidence.

### 2.7. Data Synthesis

Given the heterogeneity of intervention types and outcome measures, a narrative synthesis was undertaken following the framework of Popay et al. [40]. Findings were grouped by intervention type and by outcome domain (QoL, physical health and psychosocial health). Quantitative results (e.g., pooled standardised mean differences (SMDs)) from included meta-analyses were extracted verbatim to illustrate the magnitude and direction of effects, and findings were summarised descriptively and supported by GRADE evidence tables. For each intervention–outcome domain, greater interpretive weight was given to the most recent, comprehensive and methodologically robust review, based on its AMSTAR-2 rating, comprehensiveness of search, number of included primary studies and relevance to breast cancer survivorship. In addition, the extent to which each review reported determinants of real-world effectiveness (engagement or adherence, attrition, long-term follow-up, system integration, scalability, economic data and equity considerations) was charted as extensively reported, partially reported or absent.

## 3. Results

### 3.1. Study Selection

**Figure 1:**
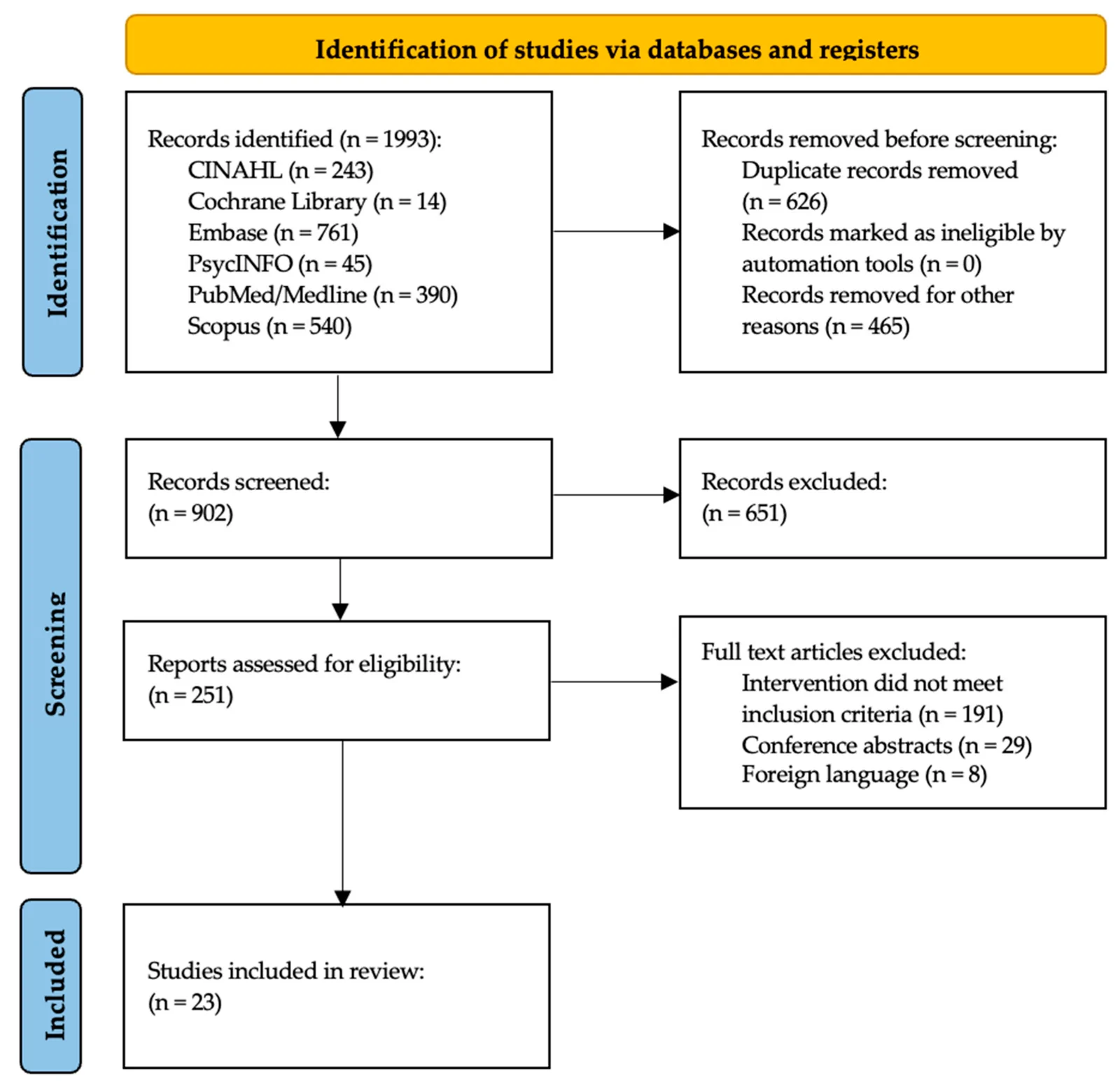
PRISMA 2020 flow diagram of the identification, screening and inclusion of systematic reviews.

A total of 1993 records were retrieved from the six databases (Figure 1). After removal of duplicates and records excluded for other reasons, 902 records underwent title and abstract screening, 251 reports were assessed for eligibility and 23 systematic reviews or meta-analyses were included [41,42,43,44,45,46,47,48,49,50,51,52,53,54,55,56,57,58,59,60,61,62,63]. These 23 reviews encompassed 459 primary study occurrences. Not all reviews reported the total sample size; the 15 reviews that did so included a combined 44,560 participants.

### 3.2. Characteristics of Included Reviews

The characteristics of the included reviews are summarised in Table S1. Of the 23 reviews, 17 (74%) were published within the past five years. The number of primary studies per review ranged from 4 [57] to 56 [53]. Twelve reviews (52%) included only RCTs, while the remaining 11 (48%) included a mix of RCTs and other designs (e.g., quasi-experimental studies). All reviews compared the intervention with usual care or another form of control.

The interventions fell into six categories: online or web-based interventions (including internet-based programmes, websites, forums and email); mobile application (mHealth) interventions; telephone-based interventions; wearable activity trackers; VR-based interventions; and AI chatbots (Table 1). Many reviews covered more than one category: 13 reviews (57%) included online interventions, 14 (61%) mHealth, 9 (39%) telephone-based interventions, 6 (26%) wearable activity trackers, 2 (9%) VR-based interventions and 1 (4%) an AI chatbot. A GRADE summary of the evidence by intervention type is provided in Table S3.

### 3.3. Methodological Quality

The AMSTAR-2 appraisal is summarised in Table 2. Overall confidence was rated low for 19 (83%) of the 23 reviews, moderate for 1 (4%) and high for 3 (13%). Most or all reviews defined the population, intervention, comparator and outcome (PICO) components (23 of 23, 100%), justified their choice of study designs (21 of 23, 91%), conducted a comprehensive search (22 of 23, 96%), listed excluded studies (22 of 23, 96%), described the included studies adequately (23 of 23, 100%), assessed risk of bias (19 of 23, 83%) and reported conflicts of interest (23 of 23, 100%). Other domains were rated less favourably: only 11 reviews (48%) registered a protocol, 17 (74%) performed study selection and data extraction in duplicate, 13 (57%) considered risk of bias when interpreting results, 17 (74%) explained heterogeneity, 6 (26%) investigated publication bias and 9 (39%) reported the funding sources of included studies. Of the 11 reviews (48%) that conducted a meta-analysis, 10 (91%) used appropriate statistical methods and 7 (64%) accounted for risk of bias in the meta-analysis.

### 3.4. Effects by Type of Intervention

The 13 reviews (57%) that included online interventions reported improved QoL [41,46,49,52,53,54,56,57,61,63], improved physical outcomes [43,49,52,53,54,56,57,63] and improved psychosocial outcomes [41,46,49,50,52,53,54,57,60,61], generally with small-to-moderate effect sizes. Physical outcomes included weight change (SMD −0.48) [53], duration of physical activity (SMD 0.71) [56], shoulder range of motion (ROM) [63], fatigue (SMD −1.23) [61], upper-extremity disability (SMD −0.96) [63] and pain (SMD −0.58) [63]. Psychosocial outcomes included anxiety, with effect sizes from −0.09 to −0.71 [53,61], and depression, with effect sizes from −0.15 to −1.27 [53,60].

The 14 reviews (61%) that included mHealth interventions reported improved QoL [45,46,49,52,53,54,56,57,58,61,62,63], improved physical outcomes [45,49,52,53,54,56,57,58,62,63], improved psychosocial outcomes [45,46,49,52,53,54,55,57,58,60,61], improved care management [45,46] and improved survivorship [45]. The physical and psychosocial outcomes reported for mHealth largely overlapped with those of online interventions, reflecting the shared evidence base. Jongerius et al. noted one primary study in which educational material about surgical treatment increased anxiety [45]; however, this finding related to the pre-treatment phase rather than to survivorship. Care management functions, such as monitoring of post-treatment side effects, personal data management, psychosocial support and post-treatment educational content, were reported positively by Jongerius et al. and Triberti et al. [45,46].

The nine reviews (39%) that included telephone-based interventions reported improved QoL [42,44,49,53,56,57,63], improved physical outcomes [43,44,49,53,57,63] and improved psychosocial outcomes [42,43,44,49,53,57,60]. Again, these outcomes largely overlapped with those of online and mHealth interventions because several reviews covered multiple modalities. Notably, Goode et al. reported that telephone-delivered interventions initiated behaviour change leading to improved physical outcomes, although maintenance of change and cost-effectiveness were not demonstrated [43].

The six reviews (26%) that included wearable activity trackers consistently reported improved QoL [51,53,63] and improved physical outcomes [47,51,53,56,59,63], including total physical activity [51,56], moderate-to-vigorous physical activity (MVPA) [51,59], daily steps [51], weight change [53,59], ROM [63], fatigue [51] and pain [63], with small-to-moderate effect sizes. Only two reviews reported improved psychosocial outcomes with wearables [47,53].

The two reviews (9%) that included VR-based interventions reported marked improvements in shoulder ROM, including flexion (SMD 1.79–2.08), extension (SMD 1.54–1.74), abduction (SMD 2.32–17.53), adduction (SMD 2.09–15.98), internal rotation (SMD 2.98–7.12) and external rotation (SMD 0.96–2.29) [48,63]. Bu et al. also reported reduced anxiety (SMD −6.47), reduced depression (SMD −4.27) and improved cognitive function (SMD 8.80), although these very large pooled estimates were derived from few small trials and were rated as having very low certainty [48]. Finally, the single review (4%) that included an AI chatbot reported improved QoL, physical and psychosocial outcomes [54].

### 3.5. Effects by Outcome Domain

Outcomes were grouped into four domains: QoL, physical, psychosocial and other outcomes (Table 3). QoL improved consistently, with pooled effect sizes ranging from 0.45 (95% confidence interval (CI) 0.22–0.69) to 1.83 (95% CI 0.44–3.22) [56,63]. Physical outcomes were divided into six sub-categories (anthropometry, physical activity, physical function, physical conditions, diet and nutrition, and behaviour change) and psychosocial outcomes into three (mental health conditions, socio-emotional well-being and mental function).

For anthropometric outcomes, reviews reported reduced body mass index (BMI) [47,56,59,62] and reduced weight [45,47,53,59]. For physical activity, reviews reported increased total physical activity [49,51,52,53,56,57,58,63], increased MVPA [47,51,59,63], increased daily steps [51,59], increased aerobic fitness [51] and reduced sedentary time [47,59,63]. For physical function, reviews reported improved function and functional capacity across physical, physiological and sexual domains [44,49,50,51,53,56,58], increased shoulder ROM [48,49,63], improved upper-extremity function [49,52,58,63] and improved cardiovascular biomarkers [47]. For physical conditions, reviews reported reduced fatigue [47,50,51,54,57,61], reduced pain and symptom burden [48,49,54,63] and improved sleep quality, with longer sleep time and less insomnia [49,50,53,57]. Diet adherence and nutritional quality also improved [53,62], as did positive behaviour change regarding weight control, diet and physical activity [43].

For psychosocial outcomes, anxiety [42,44,46,47,48,52,53,54,55,57,58,60,61] and depression [42,44,46,47,48,53,54,55,57,58,60,61] were reduced across numerous reviews. Reviews also reported reduced fear of recurrence [54,55,57,58], improved emotional well-being [42,47,52,53,57], reduced stress [41,45,46,50,52,53], increased social support [41,49,58], increased self-efficacy [44,50,52,54,55], reduced negative mood [50], improved cognitive function [47,48,57] and improved memory [47,57]. Finally, reviews reported improved care management and coordination [45,46], improved survivorship [45] and improved access to information [41,49]. There was, however, insufficient evidence on the maintenance of effects and the cost-effectiveness of the interventions [43].

### 3.6. Overlap of Primary Studies

**Figure 2:**
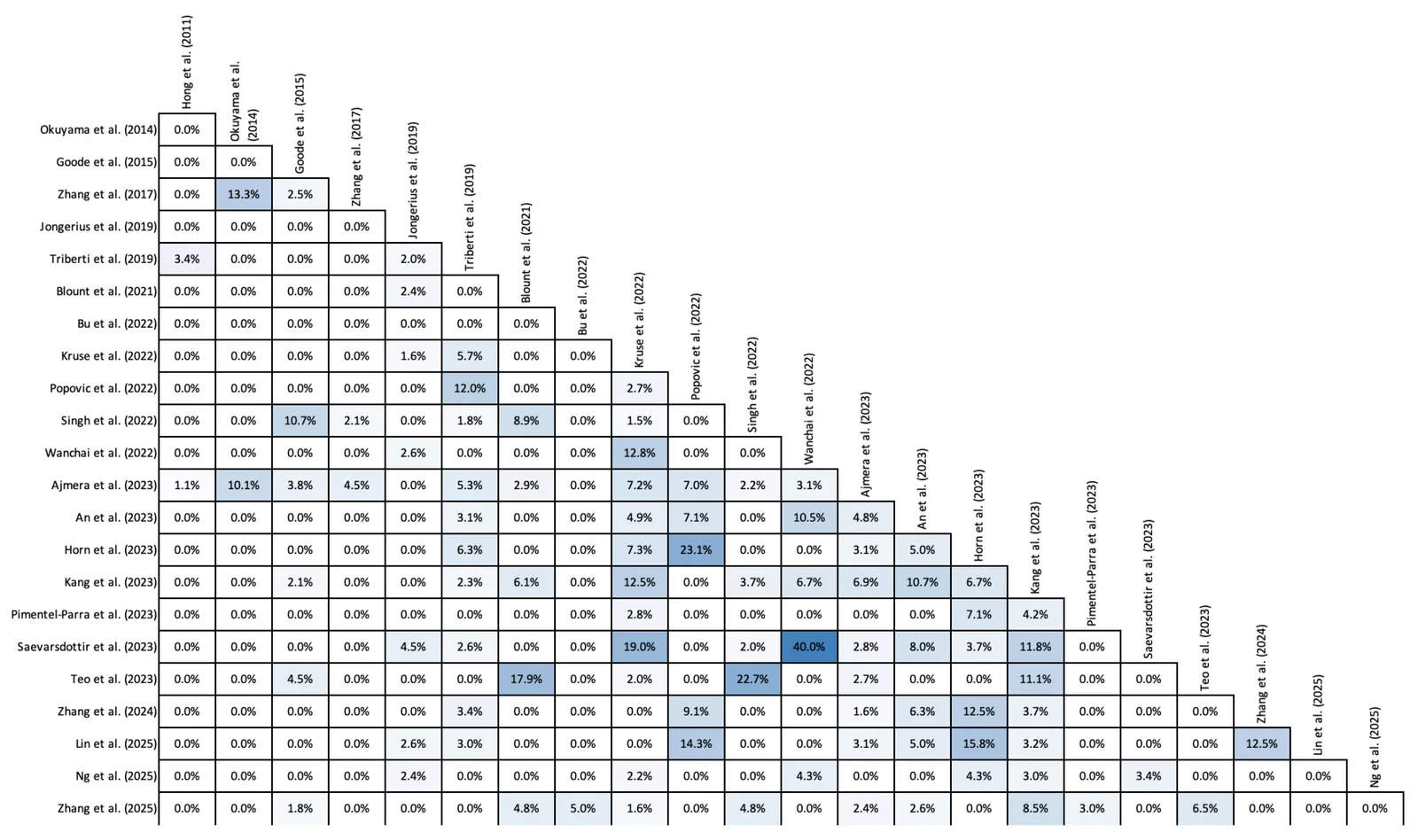
Pairwise corrected covered area (CCA) matrix of the 23 included reviews. Darker shading indicates greater overlap of primary studies between pairs of reviews. Referenced studies: Hong et al. (2011) [41]; Okuyama et al. (2014) [42]; Goode et al. (2015) [43]; Zhang et al. (2017) [44]; Jongerius et al. (2019) [45]; Triberti et al. (2019) [46]; Blount et al. (2021) [47]; Bu et al. (2022) [48]; Kruse et al. (2022) [49]; Popovic et al. (2022) [50]; Singh et al. (2022) [51]; Wanchai et al. (2022) [52]; Ajmera et al. (2023) [53]; An et al. (2023) [54]; Horn et al. (2023) [55]; Kang et al. (2023) [56]; Pimentel-Parra et al. (2023) [57]; Saevarsdottir et al. (2023) [58]; Teo et al. (2023) [59]; Zhang et al. (2024) [60]; Lin et al. (2025) [61]; Ng et al. (2025) [62]; Zhang et al. (2025) [63].

The CCA matrix in Figure 2 illustrates the degree of overlap in primary studies across the 23 reviews (full citation matrix in Table S4). In total, 333 unique primary studies accounted for the 459 study occurrences. The covered area (N/rc) was 5.99% and the CCA was 1.72%, which is interpreted as slight overlap overall. A total of 261 primary studies appeared in only one review, 41 appeared in two reviews, 17 in three, 9 in four, 1 in five and 4 in six reviews. No primary study appeared in more than six reviews. The highest pairwise overlap was between Saevarsdottir et al. [58] and Wanchai et al. [52] (40.0%), both of which focused on mHealth applications.

### 3.7. Reporting of Engagement, Integration and Scalability

**Figure 3:**
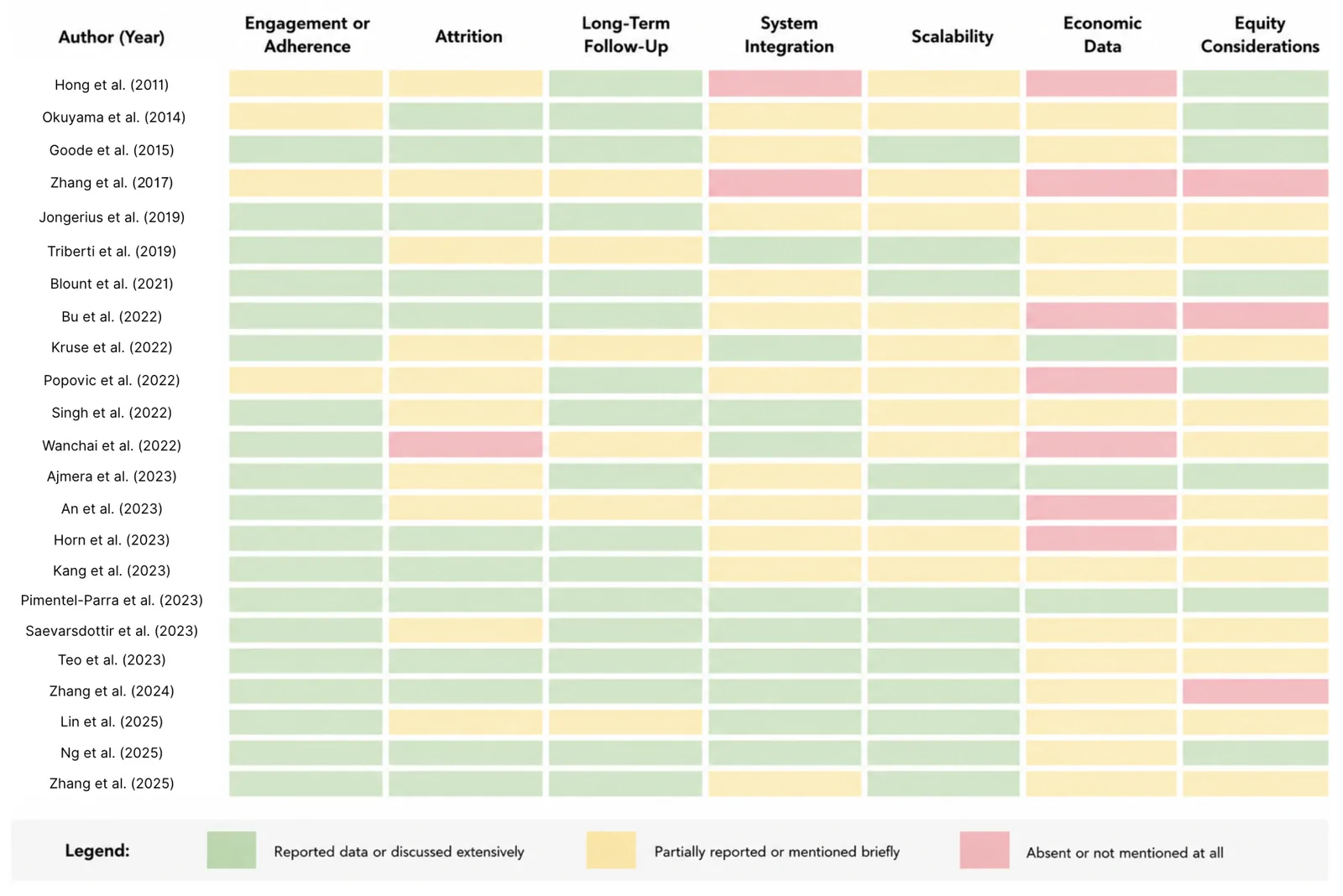
Reporting of determinants of real-world effectiveness across the 23 included reviews. Green: reported or discussed extensively; yellow: partially reported or mentioned briefly; red: absent or not mentioned. Referenced studies: Hong et al. (2011) [41]; Okuyama et al. (2014) [42]; Goode et al. (2015) [43]; Zhang et al. (2017) [44]; Jongerius et al. (2019) [45]; Triberti et al. (2019) [46]; Blount et al. (2021) [47]; Bu et al. (2022) [48]; Kruse et al. (2022) [49]; Popovic et al. (2022) [50]; Singh et al. (2022) [51]; Wanchai et al. (2022) [52]; Ajmera et al. (2023) [53]; An et al. (2023) [54]; Horn et al. (2023) [55]; Kang et al. (2023) [56]; Pimentel-Parra et al. (2023) [57]; Saevarsdottir et al. (2023) [58]; Teo et al. (2023) [59]; Zhang et al. (2024) [60]; Lin et al. (2025) [61]; Ng et al. (2025) [62]; Zhang et al. (2025) [63].

Reporting of the determinants of real-world effectiveness was heterogeneous across the 23 reviews (Figure 3; underlying data in Table S6). Engagement or adherence and long-term follow-up were well reported, by 19 (83%) and 17 (74%) reviews, respectively. Attrition was reported in 12 reviews (52%), system integration (e.g., linkage with electronic medical records or embedding within care pathways) in 10 (43%) and scalability in 12 (52%). By contrast, economic or resource-use data were reported in only three reviews (13%) and equity considerations (e.g., older, rural or low-socioeconomic-status participants) in eight (35%).

## 4. Discussion

### 4.1. Principal Findings

This umbrella review synthesises the evidence from 23 systematic reviews and meta-analyses on digital health interventions for breast cancer survivors. Although umbrella reviews with related objectives have been published [64,65,66,67,68], to our knowledge this is the broadest to date, encompassing all modalities of digital health intervention, including online, mHealth, telephone-based, wearable, VR-based and AI-based tools, and reporting on a wider array of outcomes than previous overviews [66,67,68].

Overall, digital health interventions had positive effects on QoL and on physical and psychosocial outcomes, consistent with the findings of related overviews [64,65,66,67,68]. For instance, Sabyr et al., in a 2026 umbrella review, found strong evidence that mHealth interventions produce short-term improvements in engagement, adherence, symptom management and psychosocial outcomes among breast cancer patients [64]. Our findings extend this conclusion across the full range of digital modalities and to the survivorship phase specifically, while also showing that the included reviews are of predominantly low methodological quality and that the evidence on real-world implementation is sparse.

### 4.2. Mechanisms of Benefit

Several mechanisms have been proposed for these benefits. Digital health interventions are a convenient means of enhancing patients’ ‘information competence’, that is, their ability to recognise when information is needed and to locate, evaluate, synthesise and use it effectively [69]. Kim et al. found that after six weeks of using an e-health intervention, improved information competence led to substantial reductions in information overload and to improvements in emotional functioning and social support at three months [69]. The capacity of these tools to help patients find, understand and use cancer-related information therefore appears to be a key mechanism for improving outcomes.

Moreover, digital interventions typically take a multi-component approach to promoting behaviour change, which is often the proximal driver of physical, psychosocial and QoL outcomes. The most effective interventions combine videos, forums, electronic reminders and interactive support features, provide disease- or treatment-focused information and incorporate behaviour change techniques such as self-monitoring, goal setting, problem solving and tailored feedback [70]. These features capitalise on the adaptability of digital platforms, in contrast with print or in-person interventions, which struggle to cover as many components simultaneously. In turn, this improves patients’ self-efficacy, empowering them to take charge of their own health through better decision-making and sustained behaviour change [21,25]. Our extraction of behaviour change techniques across the included reviews (Table S5) supports this interpretation: self-monitoring, goal setting and feedback on behaviour were the most commonly reported techniques across modalities.

Because all digital health modalities appear to act through similar mechanisms to elicit behaviour change, the category of intervention may have a limited relationship with the magnitude of effect on any given outcome [71]. For example, web-based and mHealth interventions commonly provide structured health education, self-management resources, and psychological support, potentially improving symptom awareness, self-efficacy, and perceived control over health. Wearable activity trackers provide feedback on physical activity and may reinforce behavioural goals through self-monitoring and progress tracking. Telephone-based interventions offer direct interpersonal support and continuity of care without requiring advanced digital skills. VR-based interventions may facilitate engagement with rehabilitation exercises through interactive environments.

This is reflected in the present review and in others, where the direction of effect is consistent across modalities and reviews [72]. Furness et al., for example, found insufficient evidence to determine whether synchronous or asynchronous digital interventions yielded superior outcomes [73].

### 4.3. Long-Term Engagement

A key limitation of the current evidence base is the limited evidence on long-term engagement. Among the included reviews, Horn et al. found limited evidence of sustained effects [55], and Zhang et al. found smaller effect sizes for long-term than for short-term outcomes [60]. Other studies and reviews have similarly reported declining engagement with digital tools over time [70,72,74]. This underscores the need to redirect research towards strategies that sustain long-term engagement, such as gamification and social networking features [75,76,77], rather than focusing solely on short-term effects.

### 4.4. An Integration Ladder for Digital Survivorship Interventions

**Figure 4:**
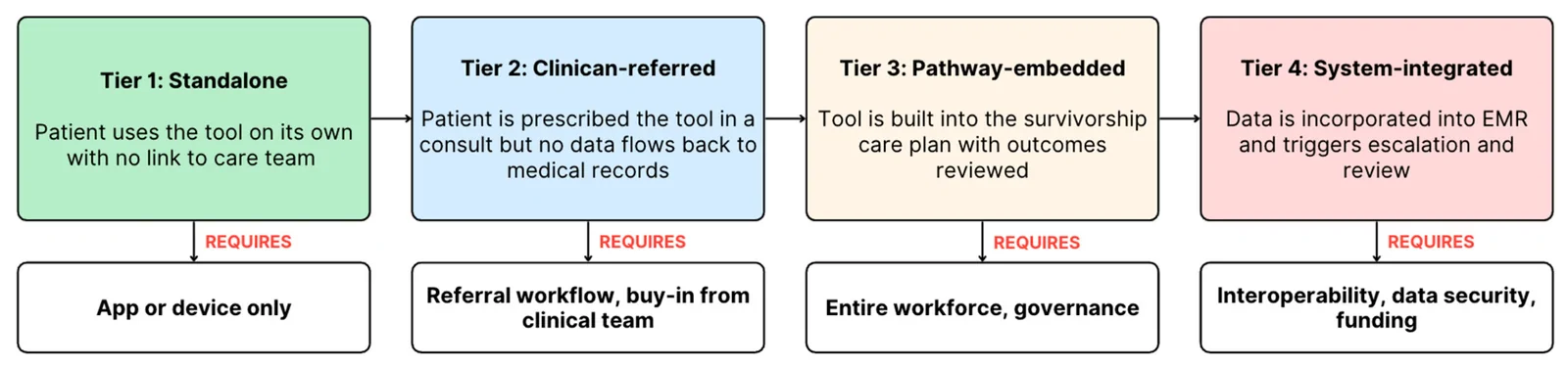
Proposed four-tier integration ladder for digital health interventions in breast cancer survivorship care, with the requirements at each tier. EMR: electronic medical record.

There is also a consistent gap between the tier of integration at which digital health interventions are *evaluated* and the tier that their effectiveness and scalability claims *presuppose*. We propose a four-tier integration ladder, comprising (1) standalone, (2) clinician-referred, (3) pathway-embedded and (4) system-integrated interventions, as a framework for situating both the existing evidence and the work still required (Figure 4). Although some reviews touch on Tiers 2 to 4, the evidence base sits predominantly at Tier 1, where the patient uses the tool independently of the care team and the intervention is evaluated on its own. Yet claims that these interventions are ‘scalable’ or ready for survivorship pathways implicitly invoke the upper tiers, which carry distinct and largely unevaluated requirements: referral workflows and clinician engagement at Tier 2; workforce capacity and governance at Tier 3; and interoperability, data governance and sustained funding at Tier 4. Until interventions are tested at the tier at which they are intended to operate, effectiveness demonstrated in standalone trials cannot be assumed to transfer.

Potential harms and practical drawbacks also differ by modality. Information-heavy apps may increase distress, and VR can cause physical discomfort such as nausea or dizziness [45,48]. Repeated prompts or symptom monitoring may be burdensome; wearables require charging and sustained use, while telephone support requires staff time and appointments. Privacy breaches, inaccurate automated advice and unclear responsibility for responding to alerts are potential implementation risks, not event rates established by this review. Programmes should therefore specify content review, data protection, clinical escalation and alternatives for patients who disengage. The available synthesis does not permit reliable comparisons of harm rates across modalities.

Translating this proposed integration ladder into clinical practice also requires consideration of several operational and equity-related factors. At lower levels of integration, clear referral and onboarding pathways, accessible interfaces, digital literacy support, and language adaptation are important to facilitate uptake among diverse survivor populations. At higher levels, interoperability with electronic medical records, clearly defined responsibilities for reviewing patient-generated data, and escalation protocols for clinically significant symptoms become increasingly important. Health systems must also address broader implementation challenges, including data privacy and governance, clinician workload, reimbursement mechanisms, and ongoing technical maintenance, as has been encountered with other digital health and AI innovations [78]. Particular attention should be given to older adults, rural populations, and survivors from lower-income backgrounds, who may face disproportionate barriers to digital access. These considerations should inform prospective implementation studies rather than be assumed to be resolved by technological availability alone.

### 4.5. Strengths and Limitations

The strengths of this review include its prospective registration, comprehensive search of six databases, duplicate screening and extraction, formal appraisal of methodological quality and certainty of evidence, quantification of primary study overlap and systematic charting of implementation-relevant reporting. Several limitations should nonetheless be acknowledged. First, outcomes were reported heterogeneously: although many reviews conducted meta-analyses and reported effect sizes with 95% confidence intervals, those that used a purely narrative approach did not, so the synthesis in this review was necessarily narrative as well. Second, only English-language reviews were included, and primary studies from high-income countries were over-represented. Although digital health tools are reasonably accessible in high-income countries [79], LMICs may not be able to adopt them with similar ease owing to infrastructure deficits [80,81], limited digital literacy [82], inadequate health system integration [83] and resource constraints that undermine implementation and sustained use [84].

Third, as an umbrella review, this study is dependent on the quality and reporting of the included reviews and could not re-analyse primary data. Consequently, we could not examine how the effectiveness and acceptability of digital health interventions vary according to patient characteristics, including age, cancer stage, treatment phase, and geographical or sociocultural context. Older survivors may encounter greater digital literacy or accessibility barriers [81], while differences in healthcare infrastructure, language, and cultural preferences may influence intervention uptake across countries [83,84]. Similarly, patients with different disease stages or treatment-related symptom burdens may have distinct supportive care needs.

A further limitation concerns the inclusion of reviews involving mixed treatment-phase populations. This broadening of eligibility introduced heterogeneity in the populations represented and may have affected the applicability of findings to post-treatment survivorship specifically. In particular, supportive care needs, symptom burden, and responsiveness to digital interventions may differ between individuals undergoing active treatment and those who have completed primary treatment. Furthermore, because many reviews did not report findings separately by treatment phase, the relative contribution of each population could not be determined.

In addition, most included reviews were rated as being of low confidence on AMSTAR-2 despite attempting a robust methodology. Common limitations included inadequate consideration of risk of bias when interpreting findings, limited assessment of publication bias, and incomplete reporting of methodological safeguards. These limitations reduce confidence in the robustness of review-level conclusions, even where individual pooled estimates suggest beneficial effects.

Finally, the overlap in primary studies, although slight overall, was substantial between certain pairs of reviews. In particular, Saevarsdottir et al. [58] and Wanchai et al. [52] shared 40.0% of their primary evidence. Consequently, the apparent consistency of findings across multiple reviews, particularly those evaluating mHealth interventions, may partly reflect repeated synthesis of the same primary studies rather than independent replication of beneficial effects. This should be considered when interpreting the overall strength of evidence.

### 4.6. Implications for Practice and Research

For clinicians and breast cancer survivors, the key message is that digital health interventions, ranging from web-based, telephone-based and mobile application tools to wearable activity trackers and AI chatbots, appear to improve QoL, physical outcomes such as physical activity and weight control, and psychosocial outcomes such as anxiety and depression. Given the growing number of cancer survivors worldwide, these interventions hold promise for improving post-treatment care while reducing the financial and logistical burden on health systems [34].

Future research should investigate the long-term engagement and sustainability of digital health tools and should measure and report engagement, adherence and attrition as standard. With the rapid development of AI, applications beyond chatbots, such as personalised symptom prediction and adaptive content delivery, merit evaluation in survivorship care. Multi-component platforms that are integrated with health systems should be developed and evaluated at the tier at which they are intended to operate, alongside the economic and implementation evidence that pathway integration requires. Lastly, as mobile devices become increasingly ubiquitous worldwide, more studies should be conducted in LMICs to evaluate the utility of mHealth tools as supportive care interventions; given that many communities continue to lack access to adequate healthcare, digital health interventions may help to bridge this gap.

## 5. Conclusions

Digital health interventions generally have favourable effects on quality of life, physical activity, and psychosocial outcomes among individuals affected by breast cancer, although the magnitude and consistency of these benefits vary across intervention modalities and outcomes. Web-based and mHealth interventions have the broadest evidence base, potentially supporting self-management through structured education, symptom monitoring, behavioural reinforcement, and psychological support. Telephone-based interventions offer an accessible alternative that may help reduce digital barriers, while wearable activity trackers appear particularly relevant for promoting physical activity and behavioural change. Virtual reality interventions show promise for rehabilitation and symptom management, although the certainty of evidence remains limited; evidence supporting AI chatbots is still preliminary.

These findings should be interpreted cautiously, as 83% of included reviews were rated as having low methodological confidence, and some reviews shared substantial primary evidence. Furthermore, evidence regarding sustained engagement, adverse effects, digital equity, economic value, and integration into routine survivorship pathways remains insufficient. Future research should prioritise rigorous, longer-term evaluations, assess potential benefits and unintended consequences across diverse patient populations, and generate implementation evidence appropriate to the intended level of health-system integration. Digital health interventions represent promising components of breast cancer supportive care, but their broader adoption should be guided by intervention-specific evidence, patient needs, and demonstrated feasibility within clinical practice.

## Figures and Tables

**Table 1:** Types of digital health intervention represented across the included reviews.

Author (Year) [Ref.]	Online or Web-Based	Mobile Application (mHealth)	Telephone-Based	Wearable Activity Tracker	VR-Based	AI Chatbot
Hong et al. (2011) [41]	✓ Support groups, forums, mailing lists, general internet use					
Okuyama et al. (2014) [42]			✓ Psychosocial counselling (e.g., CBT, interpersonal therapy, uncertainty management)			
Goode et al. (2015) [43]	✓		✓			
Zhang et al. (2017) [44]			✓			
Jongerius et al. (2019) [45]		✓				
Triberti et al. (2019) [46]	✓ Support groups, websites	✓				
Blount et al. (2021) [47]				✓		
Bu et al. (2022) [48]					✓	
Kruse et al. (2022) [49]	✓	✓	✓			
Popovic et al. (2022) [50]	✓ CBT-based modules, stress management, insomnia programmes, sexual dysfunction support					
Singh et al. (2022) [51]				✓		
Wanchai et al. (2022) [52]	✓ For side effects of treatment	✓ For side effects of treatment				
Ajmera et al. (2023) [53]	✓	✓	✓	✓		
An et al. (2023) [54]	✓	✓				✓
Horn et al. (2023) [55]		✓ CBT-based psychological support				
Kang et al. (2023) [56]	✓ Email, websites	✓	✓	✓		
Pimentel-Parra et al. (2023) [57]	✓	✓	✓			
Saevarsdottir et al. (2023) [58]		✓				
Teo et al. (2023) [59]				✓		
Zhang et al. (2024) [60]	✓ On CBT-based psychological support	✓ On CBT-based psychological support	✓ On CBT-based psychological support			
Lin et al. (2025) [61]	✓ On CBT-based psychological support	✓ On CBT-based psychological support				
Ng et al. (2025) [62]		✓				
Zhang et al. (2025) [63]	✓ On behavioural change	✓ On behavioural change	✓ On behavioural change	✓	✓ On behavioural change	

✓ (shaded) indicates that the review included the intervention type; where the review focused on a specific application, it is described in the cell. AI, artificial intelligence; CBT, cognitive behavioural therapy; mHealth, mobile health; VR, virtual reality.

**Table 2:** AMSTAR-2 methodological quality appraisal of the included reviews.

AMSTAR-2 Item	[41]	[42]	[43]	[44]	[45]	[46]	[47]	[48]	[49]	[50]	[51]	[52]	[53]	[54]	[55]	[56]	[57]	[58]	[59]	[60]	[61]	[62]	[63]
1. PICO components defined	Y	Y	Y	Y	Y	Y	Y	Y	Y	Y	Y	Y	Y	Y	Y	Y	Y	Y	Y	Y	Y	Y	Y
2. Protocol registered	N	N	N	N	Y	N	N	Y	Y	N	Y	N	N	N	Y	N	Y	N	Y	Y	Y	Y	Y
3. Justification for study design	Y	PY	Y	Y	Y	Y	N	Y	Y	Y	Y	Y	Y	Y	Y	Y	Y	N	Y	Y	PY	PY	Y
4. Comprehensive search	PY	N	Y	PY	PY	PY	PY	Y	PY	PY	PY	PY	PY	PY	Y	Y	Y	PY	Y	Y	Y	PY	Y
5. Study selection in duplicate	N	N	Y	N	N	N	PY	Y	Y	N	PY	Y	PY	Y	Y	Y	Y	Y	Y	Y	Y	Y	Y
6. Data extraction in duplicate	N	N	Y	Y	Y	N	N	Y	Y	Y	PY	Y	Y	Y	Y	N	N	Y	Y	Y	Y	PY	Y
7. List of excluded studies	Y	Y	Y	Y	Y	Y	N	Y	Y	Y	Y	Y	Y	Y	Y	Y	Y	Y	Y	Y	Y	Y	Y
8. Adequate description of included studies	Y	Y	Y	Y	Y	Y	Y	Y	Y	Y	Y	Y	Y	Y	Y	Y	Y	Y	Y	Y	Y	Y	Y
9. Risk of bias (RoB) assessment	N	PY	Y	N	PY	N	PY	Y	Y	N	PY	Y	Y	Y	Y	Y	Y	Y	Y	Y	Y	Y	Y
10. Sources of funding	N	N	N	N	N	N	Y	N	Y	N	N	Y	N	Y	Y	N	Y	N	Y	Y	N	N	Y
11. Methods for meta-analysis appropriate	NA	NA	PY	NA	NA	NA	PY	PY	NA	N	PY	NA	Y	NA	NA	Y	NA	NA	Y	Y	Y	NA	Y
12. RoB assessed for meta-analysis	NA	NA	PY	NA	NA	NA	N	PY	NA	N	N	NA	PY	NA	NA	PY	NA	NA	PY	N	PY	NA	Y
13. RoB in interpretation	N	N	PY	N	N	N	PY	Y	Y	N	PY	PY	PY	N	PY	PY	Y	N	N	N	PY	Y	Y
14. Heterogeneity explained	N	N	Y	Y	PY	N	PY	Y	N	PY	PY	Y	PY	N	Y	Y	N	PY	Y	PY	Y	Y	Y
15. Investigated publication bias	NA	N	PY	N	N	NA	N	PY	N	N	N	NA	N	N	N	PY	NA	N	Y	PY	N	N	PY
16. Conflict of interest reported	Y	Y	Y	Y	Y	Y	Y	Y	Y	Y	Y	Y	Y	Y	Y	Y	Y	PY	Y	Y	Y	Y	Y
**Overall confidence**	**Low**	**Low**	**Low**	**Low**	**Low**	**Low**	**Low**	**High**	**Low**	**Low**	**Low**	**Low**	**Low**	**Low**	**Low**	**Low**	**Low**	**Mod.**	**Low**	**High**	**Low**	**Low**	**High**

Columns are identified by reference number: Hong et al. (2011) [41]; Okuyama et al. (2014) [42]; Goode et al. (2015) [43]; Zhang et al. (2017) [44]; Jongerius et al. (2019) [45]; Triberti et al. (2019) [46]; Blount et al. (2021) [47]; Bu et al. (2022) [48]; Kruse et al. (2022) [49]; Popovic et al. (2022) [50]; Singh et al. (2022) [51]; Wanchai et al. (2022) [52]; Ajmera et al. (2023) [53]; An et al. (2023) [54]; Horn et al. (2023) [55]; Kang et al. (2023) [56]; Pimentel-Parra et al. (2023) [57]; Saevarsdottir et al. (2023) [58]; Teo et al. (2023) [59]; Zhang et al. (2024) [60]; Lin et al. (2025) [61]; Ng et al. (2025) [62]; Zhang et al. (2025) [63]. Y (green), yes; PY (yellow), partial yes; N (red), no; NA, not applicable (no meta-analysis conducted). Items 2, 4, 7, 9, 11, 13 and 15 are critical domains. Overall confidence (Low, Mod. (moderate), High) is rated as high, moderate, low or critically low according to AMSTAR-2 guidance [38].

**Table 3:** Summary of evidence by outcome domain across the included reviews.

Domain/Sub-Category	Outcome	Effect Size (Reported or Pooled)	Direction of Effect	Reviews [Ref.]
QoL				
QoL	Change in QoL	+0.45 (95% CI: 0.22–0.69) to +1.83 (95% CI: 0.44–3.22)	↑ QoL	[41,42,44,45,46,49,51,52,53,54,56,57,58,61,62,63]
Physical outcomes				
Anthropometry	BMI	Reduced	↓ BMI	[47,56,59,62]
	Weight change	Reduced	↓ weight	[45,47,53,59]
Physical activity	Total physical activity (by duration)	Increased	↑ duration	[49,51,52,53,56,57,58,63]
	Moderate-to-vigorous physical activity (MVPA) (by duration)	Increased	↑ duration	[47,51,59,63]
	Daily steps	Increased	↑ daily steps	[51,59]
	Aerobic fitness	+0.51 (95% CI: 0.28–0.74)	↑ aerobic fitness	[51]
	Sedentary time	Reduced	↓ duration	[47,59,63]
Physical function	Function and functional capacity	Increased, varied between physical, physiological, sexual function	↑ function	[44,49,50,51,53,56,58]
	Range of motion	Increased, ROM of shoulder joint	↑ ROM	[48,49,63]
	Upper-extremity function	Increased	↑ function	[49,52,58,63]
	Biomarkers	Improved, proxy for cardiovascular health	NA	[47]
Physical conditions	Fatigue	Reduced	↓ fatigue	[47,50,51,54,57,61]
	Pain and symptom burden	Reduced	↓ pain	[48,49,54,63]
	Sleep quality	Improved	↑ sleep time, ↓ insomnia	[49,50,53,57]
Diet & nutrition	Diet & nutrition	Improved	↑ diet adherence, ↑ nutrition	[53,62]
Behavioural change	Behavioural change	Improved, measured by proxy (e.g., weight control, diet control, physical activity)	↑ positive change	[43]
Psychosocial outcomes				
Mental health conditions	Anxiety	Reduced	↓ anxiety	[42,44,46,47,48,52,53,54,55,57,58,60,61]
	Depression	Reduced	↓ depression	[42,44,46,47,48,53,54,55,57,58,60,61]
Socio-emotional well-being	Fear of recurrence	Reduced	↓ fear	[54,55,57,58]
	Emotional well-being	Improved	↑ well-being	[42,47,52,53,57]
	Stress	Reduced	↓ stress	[41,45,46,50,52,53]
	Social support	Improved	↑ social support	[41,49,58]
Mental function	Self-efficacy	Improved	↑ self-efficacy	[44,50,52,54,55]
	Mood	Improved mood regulation	↓ negative mood	[50]
	Cognitive function	Improved	↑ function	[47,48,57]
	Memory	Improved	↑ memory	[47,57]
Others				
Other outcomes	Care management	Improved, easier management	↑ management	[45,46]
	Survivorship	Improved	↑ survivorship	[45]
	Access to information	Improved	↑ access	[41,49]
	Maintenance	Insufficient evidence	↔	[43]
	Cost-effectiveness	Insufficient evidence	↔	[43]

Effect sizes are standardised mean differences with 95% confidence intervals (CI) where pooled estimates were reported. BMI, body mass index; MVPA, moderate-to-vigorous physical activity; NA, not applicable; QoL, quality of life; ROM, range of motion; ↑ increase/improvement; ↓ reduction; ↔ insufficient evidence.

## Data Availability

No new data were created in this study. All data supporting the reported results are derived from previously published systematic reviews and meta-analyses cited in the reference list and are summarised in the Supplementary Materials.

## Supplementary Materials

The following supporting information can be downloaded at https://www.mdpi.com/article/10.3390/cancers18213434/s1: Table S1: Summary of the included systematic reviews and meta-analyses (n = 23); Table S2: Full search strategy for each database; Table S3: GRADE summary of evidence by type of intervention; Table S4: Citation matrix of primary studies across the 23 included reviews; Table S5: Extended data extraction of intervention design, behaviour change and implementation characteristics; Table S6: Reporting of determinants of real-world effectiveness across the included reviews. Referenced studies: Hong et al. (2011) [41]; Okuyama et al. (2014) [42]; Goode et al. (2015) [43]; Zhang et al. (2017) [44]; Jongerius et al. (2019) [45]; Triberti et al. (2019) [46]; Blount et al. (2021) [47]; Bu et al. (2022) [48]; Kruse et al. (2022) [49]; Popovic et al. (2022) [50]; Singh et al. (2022) [51]; Wanchai et al. (2022) [52]; Ajmera et al. (2023) [53]; An et al. (2023) [54]; Horn et al. (2023) [55]; Kang et al. (2023) [56]; Pimentel-Parra et al. (2023) [57]; Saevarsdottir et al. (2023) [58]; Teo et al. (2023) [59]; Zhang et al. (2024) [60]; Lin et al. (2025) [61]; Ng et al. (2025) [62]; Zhang et al. (2025) [63].

## Abbreviations

The following abbreviations are used in this manuscript:

AI	Artificial intelligence
AMSTAR-2	A MeaSurement Tool to Assess systematic Reviews, version 2
BMI	Body mass index
CBT	Cognitive behavioural therapy
CCA	Corrected covered area
CI	Confidence interval
EMR	Electronic medical record
GRADE	Grading of Recommendations, Assessment, Development and Evaluation
LMICs	Low- and middle-income countries
mHealth	Mobile health
MVPA	Moderate-to-vigorous physical activity
PRISMA	Preferred Reporting Items for Systematic Reviews and Meta-Analyses
PROSPERO	International Prospective Register of Systematic Reviews
QoL	Quality of life
RCT	Randomised controlled trial
ROM	Range of motion
SMD	Standardised mean difference
VR	Virtual reality
