# Supplementary material for: Digital Health Supportive Care Interventions for Breast Cancer Survivors: An Umbrella Review of Systematic Reviews and Meta-Analyses
Source: Cancers (Basel). 2026 Oct 23;18(21):3434. doi: 10.3390/cancers18213434 (PMC13762290; doi:10.3390/cancers18213434)
Supplement: Supplementary file 1 [file cancers-18-03434-s001.zip › cancers-4636842-supplementary.pdf]

Supplementary Materials

Table S1. Summary of the included systematic reviews and meta-analyses (n = 23).

**Author (Year) [Ref.]**	**Intervention Focus**	**No. of Studies (Sample Size)**	**Study Designs**	**Comparator**	**Study Population**	**Intervention Duration**	**Breast Cancer Representation**	**Key Outcomes**	**Main Findings**
Hong et al. (2011) [41]	Online interventions (e.g., support groups, forums, mailing lists, general internet use)	24 studies	Mixed	Usual care	Mixed (during/after treatment)	Varied, 2 to 12 months	Most studies include breast cancer	QoL, psychosocial outcomes (e.g., social support, stress), access to information	↑ QoL, ↑ psychosocial outcomes, though RCT outcomes were not statistically significant
Okuyama et al. (2014) [42]	Telephone-based interventions on psychosocial counselling (e.g., CBT, interpersonal therapy, uncertainty management)	20 studies (n = 3848)	RCT	Usual care, attention control, or telephone education	Across continuum; many newly diagnosed and survivors	Varied; some medium/long-term follow-up benefits for longer interventions	Most studies include breast cancer (13 of 20 studies)	QoL, psychosocial outcomes (e.g., emotional distress, anxiety, depression, QoL)	↑ QoL, ↑ psychosocial outcomes
Goode et al. (2015) [43]	Telephone, print, web-based interventions	27 studies (22 telephone, 3 web, 2 print)	RCT	Usual care/controls across included trials	Mixed (during/after treatment)	Varied; repeated contacts; limited long-term data	Most studies include breast cancer	Physical outcomes through behaviour change (e.g., physical activity, diet change, weight control), maintenance, cost-effectiveness	↑ physical outcomes, ↑ behaviour change, ↔ maintenance, ↔ cost-effectiveness
Zhang et al. (2016) [44]	Telephone-based interventions	14 studies (n = 2001)	RCT	Usual care, no intervention controls	Mixed (during/after treatment)	Varied, 3 weeks to 13 months	Only breast cancer	QoL, psychosocial outcomes (e.g., anxiety, depression, self-efficacy), physical outcomes	↑ QoL (SMD 0.54), ↑ psychosocial outcomes (anxiety: SMD -0.16,

Author (Year) [Ref.]	Intervention Focus	No. of Studies (Sample Size)	Study Designs	Comparator	Study Population	Intervention Duration	Breast Cancer Representation	Key Outcomes	Main Findings
								(e.g., physiological function, social-domestic function)	self-efficacy: SMD 0.22), ↑ physical outcomes (social-domestic function: SMD 0.19)
Jongerius et al. (2019) [45]	Mobile app (mHealth) interventions	29 studies	Mixed	Usual care, standard education, control apps	Across continuum; many newly diagnosed and survivors	Varied, follow-ups up to 6 months long	Only breast cancer	QoL, prevention/early detection, care management, survivorship, physical outcomes (e.g., weight loss), psychosocial outcomes (e.g., stress)	↑ QoL, ↔ prevention, ↑ care management, ↑ survivorship, conflicting on psychosocial outcomes
Triberti et al. (2019) [46]	Online (e.g., support groups, websites), mobile app (mHealth) interventions	24 studies (n = 3797)	Mixed	Usual care	Mixed (during/after treatment)	Varied, 24 weeks	Only breast cancer	QoL, care management, psychosocial outcomes (e.g., depression, stress, anxiety)	↑ QoL, ↑ care management, ↑ psychosocial outcomes
Blount et al. (2021) [47]	Wearable activity trackers	14 studies	RCT	Usual care/controls	Post-treatment survivors (≥6 months since treatment)	Varied; ≥8 weeks	Only breast cancer	Physical outcomes (e.g., physical activity, weight, BMI, biomarkers, fatigue, sedentary behaviour), psychosocial outcomes (e.g., cognitive function, memory, anxiety, depression,	↑ physical outcomes (↓ sedentary behaviour, ↑ MVPA), ↑ psychosocial outcomes

Author (Year) [Ref.]	Intervention Focus	No. of Studies (Sample Size)	Study Designs	Comparator	Study Population	Intervention Duration	Breast Cancer Representation	Key Outcomes	Main Findings
								perceived emotional well-being)	
Bu et al. (2022) [48]	VR-based interventions	12 studies	Mixed	Usual care	Survivors	Varied, 15 minutes to 10 months	Only breast cancer	Physical outcomes (e.g., ROM, pain), psychosocial outcomes (e.g., anxiety, depression, cognitive function)	↑ ROM (flexion: SMD 1.79, extension: SMD 1.54, abduction: SMD 17.53, adduction: SMD 15.98, internal rotation: SMD 7.12, external rotation: SMD 0.96), ↓ pain (SMD -1.32), ↑ psychosocial outcomes (anxiety: SMD -6.47, depression: SMD -4.27, cognitive function: SMD 8.80)
Kruse et al. (2022) [49]	Online, mobile app (mHealth), telephone-based interventions	33 studies	Mixed	Usual care, in-person care, or waitlist controls	Mixed (during/after treatment)	Varied, most are 8-12 weeks with short-term follow-up	Only breast cancer	QoL, physical outcomes, psychosocial outcomes	↑ QoL, ↑ physical outcomes, ↑ psychosocial outcomes
Popovic et al. (2022) [50]	Online interventions (e.g., CBT-based modules, stress management, insomnia programm	5 studies (n = 494)	RCT	Control/non-psychotherapy	Survivors	Varied; 4 to 16 weeks; some 1-month follow-up	Only breast cancer	Physical outcomes (e.g., insomnia, sleep quality, fatigue, sexual function), psychosocial outcomes	↑ physical outcomes, ↑ psychosocial outcomes, effect sizes 0.33-1.10

Author (Year) [Ref.]	Intervention Focus	No. of Studies (Sample Size)	Study Designs	Comparator	Study Population	Intervention Duration	Breast Cancer Representation	Key Outcomes	Main Findings
	es, sexual dysfunction support)							(e.g., distress, self-efficacy; mood)	
Singh et al. (2022) [51]	Wearable activity trackers	35 studies	RCT	Usual care or minimal intervention	Mixed (during/after treatment)	Varied, some as short as 4 weeks and others as long as 1 year	Only breast cancer	QoL, physical outcomes (e.g., physical activity, fatigue, aerobic fitness)	↑ QoL (SMD 0.26), ↑ physical outcomes, MVPA (SMD 0.61), total physical activity (SMD 0.62), daily steps (SMD 0.54), fatigue (SMD 0.18), aerobic fitness (SMD 0.51), physical function (SMD 0.66)
Wanchai et al. (2022) [52]	Online, mobile app (mHealth) interventions on side effects of treatment	11 studies (n = 1063)	Mixed	Usual care/controls, educational brochures	Mixed (during/after treatment)	Varied; some very short and some longer	Only breast cancer	QoL, physical outcomes (e.g., upper limb functionality, physical activity), psychological outcomes (e.g., self-efficacy, anxiety, stress, emotional functioning)	↑ QoL, ↑ physical outcomes, ↑ psychosocial outcomes
Ajmera et al. (2023) [53]	Telephone-based, online, mobile app (mHealth) interventio	56 studies (n = 20746)	RCT	Usual care	Survivors	Varied, longest of 4 years	Only breast cancer	QoL, physical outcomes (e.g., anthropometry, calorie intake,	↑ QoL (SMD 0.48), ↑ physical outcomes (weight

Author (Year) [Ref.]	Intervention Focus	No. of Studies (Sample Size)	Study Designs	Comparator	Study Population	Intervention Duration	Breast Cancer Representation	Key Outcomes	Main Findings
	ns, wearable activity trackers							physical activity, sexual function, sleep), psychosocial outcomes (e.g., depression, stress, anxiety, emotional well-being)	change: SMD -0.48), ↑ psychosocial outcomes (depression: SMD -1.27, stress: SMD -0.40, anxiety: SMD -0.09), but weight change and anxiety not statistically significant
An et al. (2023) [54]	Online, mobile app (mHealth) interventions, AI chatbot	10 studies	Mixed	Usual care, educational brochures	Mixed (during/after treatment)	Varied; mostly 12 weeks, some with follow-up and others immediately post	Only breast cancer	QoL, physical outcomes (e.g., pain symptoms, side effects, fatigue), psychosocial outcomes (e.g., anxiety, depression, self-efficacy, fear of recurrence)	↑ QoL, ↑ physical outcomes, ↑ psychosocial outcomes
Horn et al. (2023) [55]	Mobile app (mHealth) interventions on CBT-based psych support	11 studies (n = 2249)	RCT	Usual care, waitlist control, control apps	Survivors	Varied, 1-10 months	Only breast cancer	Psychosocial outcomes (e.g., depression, anxiety, fear of recurrence, self-efficacy)	↑ psychosocial outcomes, short-term effects with limited consistent long-term effects
Kang et al. (2023) [56]	Online (e.g., email, websites), mobile app (mHealth), telephone-based	18 studies (n = 1703)	Mixed	Usual care, non-digital exercise programmes, or NIL	Mixed (during/after treatment)	Varied, few long-term follow-ups	Only breast cancer	QoL, physical outcomes (e.g., physical activity, physical function,	↑ QoL (SMD 0.45), ↑ physical outcomes (total PA duration:

Author (Year) [Ref.]	Intervention Focus	No. of Studies (Sample Size)	Study Designs	Comparator	Study Population	Intervention Duration	Breast Cancer Representation	Key Outcomes	Main Findings
	interventions, wearable activity trackers							anthropometry)	SMD 0.71, functional capacity: SMD 0.38)
Pimentel-Parra et al. (2023) [57]	Online, mobile app (mHealth), telephone-based interventions	4 studies (n = 374)	Mixed	Usual care, no intervention controls	Survivors	Varied, 1-5 weeks	Only breast cancer	QoL, physical outcomes (e.g., vitality, physical activity, fatigue, sleep), psychosocial outcomes (e.g., cognition, memory, emotional well-being, depression, anxiety, fear of recurrence)	↑ QoL, ↑ physical outcomes, ↑ psychosocial outcomes, effect sizes 0.58-0.87
Saevarsdottir et al. (2023) [58]	Mobile app (mHealth) interventions	17 studies (n = 1657)	Mixed	Usual care	Mixed (during/after treatment)	3 to 12 weeks	Only breast cancer	QoL, physical outcomes (e.g., symptom control, physical activity, upper limb functionality), psychosocial outcomes (e.g., social support, anxiety, depression, fear of recurrence)	↑ QoL, ↑ physical outcomes, ↑ psychosocial outcomes
Teo et al. (2023) [59]	Wearable activity trackers	17 studies (n = 1293)	RCT	Usual care, waitlist care or active care	Survivors	Intervention 10 weeks to 6 months / Follow-up 24 weeks to 18 months, but majority did not	Only breast cancer	Physical outcomes (e.g., BMI, physical activity, steps per day, sedentary behaviour)	↑ MVPA (SMD 0.44, greater effect on participants with lower baseline activity), ↓

Author (Year) [Ref.]	Intervention Focus	No. of Studies (Sample Size)	Study Designs	Comparator	Study Population	Intervention Duration	Breast Cancer Representation	Key Outcomes	Main Findings
						conduct follow-up			weight (SMD -0.40), ↔ BMI
Zhang et al. (2024) [60]	Online, mobile apps (mHealth), and telephone-based interventions on CBT-based psych support	7 studies (n = 1016)	RCT	Usual care or waitlist control	Survivors	Varied; limited long-term evidence	Only breast cancer	Psychosocial outcomes (e.g., anxiety, depression)	↑ psychosocial outcomes, immediate effects (anxiety: SMD -0.14, depression: SMD -0.19) and sustained effects (anxiety: SMD -0.15, depression: SMD -0.15)
Lin et al. (2025) [61]	Online, mobile apps (mHealth) interventions on CBT-based psych support	11 studies (n = 1307)	RCT	Usual care, health ed, or waitlist control	Mixed (during/after treatment)	4-24 weeks variation, no long-term follow-up	Only breast cancer	QoL, physical outcomes (e.g., fatigue), psychosocial outcomes (e.g., anxiety, depression)	↑ QoL (SMD 0.79), ↓ fatigue (SMD -1.23), ↑ psychosocial outcomes (anxiety: SMD -0.71, depression: SMD -0.67)
Ng et al. (2025) [62]	Mobile apps (mHealth) interventions	13 studies (n = 783)	Mixed	Usual care/controls or pre-post	Mixed (patients and survivors; majority survivors)	Varied; limited long-term evidence	Most studies include breast cancer	QoL, physical outcomes (e.g., anthropometry, nutrition, symptom burden, diet)	↑ QoL, ↑ physical outcomes
Zhang et al. (2025) [63]	Online, mobile apps (mHealth), telephone-based, VR-based	29 studies (n = 2229)	RCT	Usual care, planning activities, no-intervention controls	Survivors	Varied, 2 weeks to 2 years	Only breast cancer	QoL, physical outcomes (e.g., ROM, upper-extremity disability, pain, physical	↑ QoL (SMD 1.83), ↑ ROM (flexion: SMD 2.08, extension:

Author (Year) [Ref.]	Intervention Focus	No. of Studies (Sample Size)	Study Designs	Comparator	Study Population	Intervention Duration	Breast Cancer Representation	Key Outcomes	Main Findings
	interventions on behavioural change, wearable activity trackers							activity, sedentary time)	SMD 1.74, abduction: SMD 2.32, adduction: SMD 2.09, internal rotation: SMD 2.98, external rotation: SMD 2.29), ↑ finger climbing wall height (SMD 1.65), ↓ upper-extremity disability (SMD -0.96), ↓ pain (SMD -0.58), ↔ physical activity, MVPA, sedentary time, and physical function

Abbreviations: AI, artificial intelligence; BMI, body mass index; CBT, cognitive behavioural therapy; MVPA, moderate-to-vigorous physical activity; PA, physical activity; QoL, quality of life; RCT, randomised controlled trial; ROM, range of motion; SMD, standardised mean difference; VR, virtual reality. ↑ improvement/increase; ↓ reduction; ↔ no significant effect or insufficient evidence.

Table S2. Full search strategy for each database (searched from inception to 31 July 2025).

Database	Search String
PubMed/MEDLINE	("breast cancer"[Title/Abstract] OR "breast neoplasm*"[Title/Abstract] OR "breast carcinoma"[Title/Abstract] OR "Breast Neoplasms"[MeSH]) AND ("telehealth"[Title/Abstract] OR "eHealth"[Title/Abstract] OR "digital health [Title/Abstract] OR "mobile health"[Title/Abstract] OR "mobile app*"[Title/Abstract]) AND ("cancer survivor*"[tiab] OR "breast cancer survivor*"[tiab] OR "survivorship"[tiab] OR "survivorship care"[tiab])
Embase	('breast neoplasm*':ti,ab OR 'breast carcinoma':ti,ab OR 'breast cancer':ti,ab) AND (telehealth:ti,ab OR ehealth:ti,ab OR 'digital health':ti,ab OR 'mobile health':ti,ab OR 'mobile app*':ti,ab) AND ('systematic review'/exp OR 'meta analysis'/exp OR 'systematic review':ti,ab OR 'meta-analysis':ti,ab)
CINAHL	(MH "breast cancer" OR "breast neoplasm*" OR "breast carcinoma") AND (MH telehealth OR eHealth OR "digital health" OR "mobile health" OR "mobile app*) AND (MH "Systematic Review" OR MH "Meta Analysis" OR "systematic review" OR "meta-analysis")
PsycINFO	(breast cancer or breast neoplasm* or breast carcinoma) AND (telehealth or eHealth or "digital health" or "mobile health" or "mobile app*") AND ("systematic review" OR "meta-analysis")

Database	Search String
Scopus	(TITLE-ABS-KEY("breast cancer" OR "breast neoplasm*" OR "breast carcinoma")) AND (TITLE-ABS-KEY(telehealth OR ehealth OR "digital health" OR "mobile health" OR "mobile app*")) AND (TITLE-ABS-KEY("systematic review" OR "meta-analysis"))
Cochrane Library	("breast cancer" OR "breast neoplasm*" OR "breast carcinoma") AND (telehealth OR eHealth OR "digital health" OR "mobile health" OR "mobile apps" OR "mobile app")

Search strings were adapted to the syntax of each database. Results were limited to English-language, peer-reviewed systematic reviews and meta-analyses; grey literature and conference abstracts were excluded. MeSH, Medical Subject Headings; MH, CINAHL subject heading; ti,ab, title/abstract field.

Table S3. GRADE summary of evidence by type of intervention.

Intervention Category	Summary of Evidence [Ref.]	Certainty of Evidence (GRADE)
Online, internet, web-based interventions	↑ QoL, ↑ psychosocial outcomes, though RCT outcomes were not statistically significant [41]	Very low (⊕OOO)
	↑ physical outcomes, ↑ behaviour change, ↔ maintenance, ↔ cost-effectiveness [43]	High (⊕⊕⊕⊕)
	↑ QoL, ↑ care management, ↑ psychosocial outcomes [46]	Moderate (⊕⊕⊕O)
	↑ QoL, ↑ physical outcomes, ↑ psychosocial outcomes [49]	High (⊕⊕⊕⊕)
	↑ physical outcomes, ↑ psychosocial outcomes, effect sizes 0.33-1.10 [50]	Moderate (⊕⊕⊕O)
	↑ QoL, ↑ physical outcomes, ↑ psychosocial outcomes [52]	High (⊕⊕⊕⊕)
	↑ QoL (SMD 0.48), ↑ physical outcomes (weight change: SMD -0.48), ↑ psychosocial outcomes (depression: SMD -1.27, stress: SMD -0.40, anxiety: SMD -0.09), but weight change and anxiety not statistically significant [53]	Low (⊕⊕OO)
	↑ QoL, ↑ physical outcomes, ↑ psychosocial outcomes [54]	Low (⊕⊕OO)
	↑ QoL (SMD 0.45), ↑ physical outcomes (total PA duration: SMD 0.71, functional capacity: SMD 0.38) [56]	Moderate (⊕⊕⊕O)
	↑ QoL, ↑ physical outcomes, ↑ psychosocial outcomes, effect sizes 0.58-0.87 [57]	Very low (⊕OOO)
	↑ psychosocial outcomes, immediate effects (anxiety: SMD -0.14, depression: SMD -0.19) and sustained effects (anxiety: SMD -0.15, depression: SMD -0.15) [60]	Moderate (⊕⊕⊕O)
	↑ QoL (SMD 0.79), ↓ fatigue (SMD -1.23), ↑ psychosocial outcomes (anxiety: SMD -0.71, depression: SMD -0.67) [61]	Low (⊕⊕OO)
	↑ QoL (SMD 1.83), ↑ ROM (flexion: SMD 2.08, extension: SMD 1.74, abduction: SMD 2.32, adduction: SMD 2.09, internal rotation: SMD 2.98, external rotation: SMD 2.29), ↑ finger climbing wall height (SMD 1.65), ↓ upper-extremity disability (SMD -0.96), ↓ pain (SMD -0.58), ↔ physical activity, MVPA, sedentary time, and physical function [63]	High (⊕⊕⊕⊕)
Telephone-based interventions	↑ QoL, ↑ psychosocial outcomes [42]	Moderate (⊕⊕⊕O)
	↑ physical outcomes, ↑ behaviour change, ↔ maintenance, ↔ cost-effectiveness [43]	High (⊕⊕⊕⊕)
	↑ QoL (SMD 0.54), ↑ psychosocial outcomes (anxiety: SMD -0.16, self-efficacy: SMD 0.22), ↑ physical outcomes (social-domestic function: SMD 0.19) [44]	Low (⊕⊕OO)
	↑ QoL, ↑ physical outcomes, ↑ psychosocial outcomes [49]	High (⊕⊕⊕⊕)
	↑ QoL (SMD 0.48), ↑ physical outcomes (weight change: SMD -0.48), ↑ psychosocial outcomes (depression: SMD -1.27, stress: SMD -0.40, anxiety: SMD -0.09), but weight change and anxiety not statistically significant [53]	Low (⊕⊕OO)
	↑ QoL (SMD 0.45), ↑ physical outcomes (total PA duration: SMD 0.71, functional capacity: SMD 0.38) [56]	Moderate (⊕⊕⊕O)
	↑ QoL, ↑ physical outcomes, ↑ psychosocial outcomes, effect sizes 0.58-0.87 [57]	Very low (⊕OOO)
	↑ psychosocial outcomes, immediate effects (anxiety: SMD -0.14, depression: SMD -0.19) and sustained effects (anxiety: SMD -0.15, depression: SMD -0.15) [60]	Moderate (⊕⊕⊕O)

Intervention Category	Summary of Evidence [Ref.]	Certainty of Evidence (GRADE)
	↑ QoL (SMD 1.83), ↑ ROM (flexion: SMD 2.08, extension: SMD 1.74, abduction: SMD 2.32, adduction: SMD 2.09, internal rotation: SMD 2.98, external rotation: SMD 2.29), ↑ finger climbing wall height (SMD 1.65), ↓ upper-extremity disability (SMD -0.96), ↓ pain (SMD -0.58), ↔ physical activity, MVPA, sedentary time, and physical function [63]	High (⊕⊕⊕⊕)
Mobile apps (mHealth)	↑ QoL, ↔ prevention, ↑ care management, ↑ survivorship, conflicting on psychosocial outcomes [45]	Very low (⊕OOO)
	↑ QoL, ↑ care management, ↑ psychosocial outcomes [46]	Moderate (⊕⊕⊕O)
	↑ QoL, ↑ physical outcomes, ↑ psychosocial outcomes [49]	High (⊕⊕⊕⊕)
	↑ QoL, ↑ physical outcomes, ↑ psychosocial outcomes [52]	High (⊕⊕⊕⊕)
	↑ QoL (SMD 0.48), ↑ physical outcomes (weight change: SMD -0.48), ↑ psychosocial outcomes (depression: SMD -1.27, stress: SMD -0.40, anxiety: SMD -0.09), but weight change and anxiety not statistically significant [53]	Low (⊕⊕OO)
	↑ QoL, ↑ physical outcomes, ↑ psychosocial outcomes [54]	Low (⊕⊕OO)
	↑ psychosocial outcomes, short-term effects with limited consistent long-term effects [55]	Low (⊕⊕OO)
	↑ QoL (SMD 0.45), ↑ physical outcomes (total PA duration: SMD 0.71, functional capacity: SMD 0.38) [56]	Moderate (⊕⊕⊕O)
	↑ QoL, ↑ physical outcomes, ↑ psychosocial outcomes, effect sizes 0.58-0.87 [57]	Very low (⊕OOO)
	↑ QoL, ↑ physical outcomes, ↑ psychosocial outcomes [58]	Moderate (⊕⊕⊕O)
	↑ psychosocial outcomes, immediate effects (anxiety: SMD -0.14, depression: SMD -0.19) and sustained effects (anxiety: SMD -0.15, depression: SMD -0.15) [60]	Moderate (⊕⊕⊕O)
	↑ QoL (SMD 0.79), ↓ fatigue (SMD -1.23), ↑ psychosocial outcomes (anxiety: SMD -0.71, depression: SMD -0.67) [61]	Low (⊕⊕OO)
	↑ QoL, ↑ physical outcomes [62]	Low (⊕⊕OO)
	↑ QoL (SMD 1.83), ↑ ROM (flexion: SMD 2.08, extension: SMD 1.74, abduction: SMD 2.32, adduction: SMD 2.09, internal rotation: SMD 2.98, external rotation: SMD 2.29), ↑ finger climbing wall height (SMD 1.65), ↓ upper-extremity disability (SMD -0.96), ↓ pain (SMD -0.58), ↔ physical activity, MVPA, sedentary time, and physical function [63]	High (⊕⊕⊕⊕)
Wearable activity trackers	↑ physical outcomes (↓ sedentary behaviour, ↑ MVPA), ↑ psychosocial outcomes [47]	Moderate (⊕⊕⊕O)
	↑ QoL (SMD 0.26), ↑ physical outcomes, MVPA (SMD 0.61), total physical activity (SMD 0.62), daily steps (SMD 0.54), fatigue (SMD 0.18), aerobic fitness (SMD 0.51), physical function (SMD 0.66) [51]	Low (⊕⊕OO)
	↑ QoL (SMD 0.48), ↑ physical outcomes (weight change: SMD -0.48), ↑ psychosocial outcomes (depression: SMD -1.27, stress: SMD -0.40, anxiety: SMD -0.09), but weight change and anxiety not statistically significant [53]	Low (⊕⊕OO)
	↑ QoL (SMD 0.45), ↑ physical outcomes (total PA duration: SMD 0.71, functional capacity: SMD 0.38) [56]	Moderate (⊕⊕⊕O)
	↑ MVPA (SMD 0.44, greater effect on participants with lower baseline activity), ↓ weight (SMD -0.40), ↔ BMI [59]	High (⊕⊕⊕⊕)
	↑ QoL (SMD 1.83), ↑ ROM (flexion: SMD 2.08, extension: SMD 1.74, abduction: SMD 2.32, adduction: SMD 2.09, internal rotation: SMD 2.98, external rotation: SMD 2.29), ↑ finger climbing wall height (SMD 1.65), ↓ upper-extremity disability (SMD -0.96), ↓ pain (SMD -0.58), ↔ physical activity, MVPA, sedentary time, and physical function [63]	High (⊕⊕⊕⊕)
VR-based technology	↑ ROM (flexion: SMD 1.79, extension: SMD 1.54, abduction: SMD 17.53, adduction: SMD 15.98, internal rotation: SMD 7.12, external rotation: SMD 0.96), ↓ pain (SMD -1.32), ↑ psychosocial outcomes (anxiety: SMD -6.47, depression: SMD -4.27, cognitive function: SMD 8.80) [48]	Very low (⊕OOO)

Intervention Category	Summary of Evidence [Ref.]	Certainty of Evidence (GRADE)
	↑ QoL (SMD 1.83), ↑ ROM (flexion: SMD 2.08, extension: SMD 1.74, abduction: SMD 2.32, adduction: SMD 2.09, internal rotation: SMD 2.98, external rotation: SMD 2.29), ↑ finger climbing wall height (SMD 1.65), ↓ upper-extremity disability (SMD -0.96), ↓ pain (SMD -0.58), ↔ physical activity, MVPA, sedentary time, and physical function [63]	High (⊕⊕⊕⊕)
AI chatbot	↑ QoL, ↑ physical outcomes, ↑ psychosocial outcomes [54]	Low (⊕⊕OO)

Certainty of evidence was rated by two reviewers using the GRADE approach (⊕⊕⊕⊕ high; ⊕⊕⊕O moderate; ⊕⊕OO low; ⊕OOO very low), considering risk of bias, imprecision, indirectness, inconsistency and magnitude of effect, and taking into account the GRADE ratings reported by the original reviews where available. Reviews that covered more than one intervention type appear under each relevant category. BMI, body mass index; MVPA, moderate-to-vigorous physical activity; PA, physical activity; QoL, quality of life; ROM, range of motion; SMD, standardised mean difference. ↑ improvement/increase; ↓ reduction; ↔ no significant effect or insufficient evidence.

Table S4. Citation matrix of the 333 unique primary studies across the 23 included reviews. ● indicates that the primary study was included in the review. Columns are identified by the reference number of each review: [41] Hong et al. (2011); [42] Okuyama et al. (2014); [43] Goode et al. (2015); [44] Zhang et al. (2016); [45] Jongerius et al. (2019); [46] Triberti et al. (2019); [47] Blount et al. (2021); [48] Bu et al. (2022); [49] Kruse et al. (2022); [50] Popovic et al. (2022); [51] Singh et al. (2022); [52] Wanchai et al. (2022); [53] Ajmera et al. (2023); [54] An et al. (2023); [55] Horn et al. (2023); [56] Kang et al. (2023); [57] Pimentel-Parra et al. (2023); [58] Saevarsdottir et al. (2023); [59] Teo et al. (2023); [60] Zhang et al. (2024); [61] Lin et al. (2025); [62] Ng et al. (2025); [63] Zhang et al. (2025).

Year	First Author	Title	Source	[41]	[42]	[43]	[44]	[45]	[46]	[47]	[48]	[49]	[50]	[51]	[52]	[53]	[54]	[55]	[56]	[57]	[58]	[59]	[60]	[61]	[62]	[63]
1994	Gustafson	Development and pilot evaluation of a computer-based support system for women with breast cancer	J Psychosoc Oncol. 1994;11:69–93	●																						
1995	Weinberg	Therapeutic factors: their presence in a computer-mediated support group	Soc Work Groups. 1995;18:57–69	●																						
1996	Weinberg	Online help: cancer patients participate in a computer-mediated support group	Health Soc Work. 1996;21:24–29	●																						
1997	Fernsler	Evaluation of a computer-based cancer support network	Cancer Pract. 1997;5:46–51	●																						
1998	Braden	A Self-Help Intervention for Women With Breast Cancer	Not reported in review				●																			
1999	Craddock	Self-Care Telephone Intervention for Women With Early Breast Cancer	Not reported in review				●																			
1999	Gustafson	Empowering patients using computer-based health support systems	Qual Health Care. 1999;8:49–56	●																						
1999	Klemm	Gender differences on Internet cancer support groups	Comput Nurs. 1999;17:65–72	●																						
2000	Sandgren	Telephone therapy for patients with breast cancer	Not reported in review		●																					
2000	Shaw	Experiences of women with breast cancer: exchanging social support over the CHESS computer network	J Health Commun. 2000;5:135–159	●																						
2001	Campbell	Health behavior changes after colon cancer: a comparison of face-to-face and online focus groups	Fam Community Health. 2001;24:88–103	●																						
2001	Gustafson	Effect of computer support on younger women with breast cancer	10.1046/j.1525-1497.2001.016009435.x	●					●																	
2001	Rock	Reduction in Fat Intake Is Not Associated With Weight Loss in Most Women After Breast Cancer Diagnosis: Evidence From a Randomized Controlled Trial														●										

Year	First Author	Title	Source	[41]	[42]	[43]	[44]	[45]	[46]	[47]	[48]	[49]	[50]	[51]	[52]	[53]	[54]	[55]	[56]	[57]	[58]	[59]	[60]	[61]	[62]	[63]
2002	Allen	Nurse-Led Problem-Solving Skills Training by Telephone for Women With Breast Cancer	Not reported in review				•																			
2002	Djuric	A dietary intervention to reduce body weight and adiposity in breast cancer survivors	Not reported			•																				
2002	Fogel	Internet use and social support in women with breast cancer	Health Psychol. 2002;21:398–404	•																						
2002	Klemm	Depression in Internet and face-to-face cancer support groups: a pilot study	Oncol Nurs Forum. 2002;29:E45–E51	•																						
2002	Lieberman	Self-help groups and the Internet: breast cancer newsgroups	Int J Self Help Self Care. 2002;1:323–344	•																						
2002	Mishel	Helping patients with localized prostate carcinoma manage uncertainty and treatment side effects	10.1002/cncr.10390		•																					
2002	Samarel	Effects of two types of social support and education on adaptation to early stage breast cancer	10.1002/nur.10061		•											•										
2003	Courneya	Randomized Controlled Trial of Exercise Training in Post-Surgical Colorectal Cancer Survivors	Not reported			•																				
2003	Fogel	Racial/ethnic differences and potential psychological benefits in Internet use by women with breast cancer	Psychooncology. 2003;12:107–117	•																						
2003	Heiney	Efficacy of therapeutic group by telephone for women with breast cancer	Not reported		•		•																			
2003	Lieberman	Electronic support groups for breast carcinoma: a clinical trial of effectiveness	Cancer. 2003;97:920–925	•																						
2003	Lieberman	Self-Help Online: An Outcome Evaluation of Breast Cancer Bulletin Boards	10.1348/135910705X42957						•																	
2003	Schneider	Virtual Reality as a Distraction Intervention for Women Receiving Chemotherapy for Breast Cancer	10.1016/S1078-1439(03)00173-2								•															
2003	Winzelberg	Evaluation of an Internet support group for women with primary breast cancer	Cancer. 2003;97:1164–1173	•																						
2004	Bailey	Uncertainty intervention for watchful waiting in prostate cancer	Not reported		•																					

Year	First Author	Title	Source	[41]	[42]	[43]	[44]	[45]	[46]	[47]	[48]	[49]	[50]	[51]	[52]	[53]	[54]	[55]	[56]	[57]	[58]	[59]	[60]	[61]	[62]	[63]
2004	Fogel	Internet breast health information use and coping among women with breast cancer	CyberPsychol Behav. 2004;7:59–63	•																						
2004	Pierce	Telephone Counseling Intervention Increases Intakes of Micronutrient- and Phytochemical-Rich Vegetables, Fruit and Fiber in Breast Cancer Survivors														•										
2004	Schneider	Virtual Reality as a Distraction Intervention for Older Women Receiving Chemotherapy	10.1016/j.oncn.2004.07.004								•															
2004	Winzelberg	An Internet Support Group for Women With Primary Breast Cancer														•										
2004	Wyatt	Telephone Interview of Home Nursing for Women With Breast Cancer	Not reported in review				•																			
2005	Badger	Telephone Interpersonal Counseling for Women With Breast Cancer	Not reported in review				•																			
2005	Gustafson	Use and impact of eHealth system by low-income women with breast cancer	J Health Commun. 2005;10:195–218	•																						
2005	Høybye	Online interaction: effects of storytelling in an Internet breast cancer support group	Psychooncology. 2005;14:211–220	•																						
2005	Lieberman	Self-help online: an outcome evaluation of breast cancer bulletin boards	J Health Psychol. 2005;10:855–862	•																						
2005	Mishel	Benefits from an uncertainty management intervention for African-American and Caucasian older long-term breast cancer survivors	10.1002/pon.909		•		•									•										
2005	Owen	Randomized pilot of a self-guided Internet coping group for women with early-stage breast cancer	Ann Behav Med. 2005;30:54–64	•					•							•										
2005	Pinto	Home-based physical activity intervention for breast cancer patients												•								•				
2005	Rodgers	Internet community group participation: psychosocial benefits for women with breast cancer	J Comput Mediat Commun. 2005;10(4):Article 5	•																						

Year	First Author	Title	Source	[41]	[42]	[43]	[44]	[45]	[46]	[47]	[48]	[49]	[50]	[51]	[52]	[53]	[54]	[55]	[56]	[57]	[58]	[59]	[60]	[61]	[62]	[63]
2005	Thorsen	Effectiveness of Physical Activity on Cardiorespiratory Fitness and Health-Related Quality of Life in Young and Middle-Aged Cancer Patients Shortly After Chemotherapy	Not reported			•																				
2006	Aranda	A Supportive Care Intervention for People With Cancer: Reducing Unmet Needs and Psychological Distress														•										
2006	Demark-Wahnefried	Main Outcomes of the FRESH START Trial: A Sequentially Tailored, Diet and Exercise Mailed Print Intervention Among Breast and Prostate Cancer Survivors	Not reported			•																				
2006	Lieberman	Not all negative emotions are equal: emotional expression in online support groups	Psychooncology. 2006;15:160–168	•																						
2006	Shaw	Effects of insightful disclosure within computer-mediated support groups on women with breast cancer	Health Commun. 2006;19:133–142	•																						
2007	Allard	Day surgery for breast cancer: effects of a psychoeducational telephone intervention on functional status and emotional distress	10.1188/07.ONF.133-141		•		•																			
2007	Badger	Depression and anxiety in women with breast cancer and their partners	Not reported		•																					
2007	Beaudoin	Benefiting from social capital in online support groups: an empirical study of cancer patients	CyberPsychol Behav. 2007;10:587–590	•																						
2007	Bennett	Randomized Controlled Trial of a Physical Activity Intervention for Fatigued Cancer Survivors	Not reported			•								•												
2007	Demark-Wahnefried	Long-Term Maintenance Results of the FRESH START Trial	Not reported			•																				
2007	Downe-Wamboldt	The effects and expense of augmenting usual cancer clinic care with telephone problem-solving counseling	Not reported		•																					

Year	First Author	Title	Source	[41]	[42]	[43]	[44]	[45]	[46]	[47]	[48]	[49]	[50]	[51]	[52]	[53]	[54]	[55]	[56]	[57]	[58]	[59]	[60]	[61]	[62]	[63]
2007	Gotay	Impact of a peer-delivered telephone intervention for women experiencing a breast cancer recurrence	10.1200/JCO.2006.07.4674		•											•										
2007	Lieberman	The role of insightful disclosure in outcomes for women in peer-directed breast cancer groups	Psychooncology. 2007;16:961–964	•																						
2007	Matthews	Evaluation of a 12-week home-based walking intervention for breast cancer survivors	Support Care Cancer. 2007;15(2):203–11.			•								•												
2007	Meier	How cancer survivors provide support on cancer-related Internet mailing lists	J Med Internet Res. 2007;9:e12	•																						
2007	Pierce	The Women's Healthy Eating and Living (WHEL) Study	Not reported			•										•										
2007	Sandgren	Long-term telephone therapy outcomes for breast cancer patients	10.1002/pon.1038		•		•									•										
2007	Shaw	How women with breast cancer learn using interactive cancer communication systems	Health Educ Res. 2007;22:108–119	•																						
2007	Shaw	Effects of prayer and religious expression within computer support groups	Psychooncology. 2007;16:676–687	•																						
2007	Vallance	Randomized Trial of Print Materials and Pedometers to Increase Physical Activity Among Breast Cancer Survivors	Not reported			•								•								•				
2008	Ashing-Giwa	Enhancing physical well-being and overall quality of life among underserved Latina-American cervical cancer survivors	10.1007/s11764-008-0061-2		•																					
2008	Budin	Psychosocial and Functional Outcomes of a Nursing Intervention for Newly Diagnosed Breast Cancer Patients														•										
2008	Changrani	Online cancer support groups: experiences with underserved immigrant Latinas	Prim Psychiatry. 2008;15:55–62	•																						
2008	Demark-Wahnefried	Weight Maintenance Intervention in Premenopausal Breast Cancer Patients Undergoing Chemotherapy	Not reported			•																				

Year	First Author	Title	Source	[41]	[42]	[43]	[44]	[45]	[46]	[47]	[48]	[49]	[50]	[51]	[52]	[53]	[54]	[55]	[56]	[57]	[58]	[59]	[60]	[61]	[62]	[63]
2008	Han	Expressing positive emotions within online support groups by women with breast cancer	J Health Psychol. 2008;13:1002–1007	•																						
2008	Irwin	Yale Exercise and Survivorship Study																				•				
2008	Klemm	Late effects of treatment for long-term cancer survivors: qualitative analysis of an online support group	CIN. 2008;26:49–58	•																						
2008	Nelson	Stress, immunity, and cervical cancer: biobehavioral outcomes of a randomized clinical trial	10.1158/1078-0432.CCR-07-1632		•																					
2008	Parsons	Telephone Counseling to Promote Dietary Change in Prostate Cancer Survivors	Not reported			•																				
2008	Pinto	Home-Based Physical Activity Intervention for Breast Cancer Survivors	Not reported			•																				
2008	Wise	Effects of using online narrative and didactic information on healthcare participation	Patient Educ Couns. 2008;70:348–356	•																						
2008	van Uden-Kraan	Empowering processes and outcomes of participation in online support groups	Qual Health Res. 2008;18:405–417	•																						
2009	Beaver	Comparing Hospital and Telephone Follow-Up After Treatment for Breast Cancer: Randomized Equivalence Trial														•										
2009	Campbell	Increasing Fruit and Vegetable Consumption and Physical Activity Among Colorectal Cancer Survivors	Not reported			•																				
2009	Ell	Telephone Care Management and Adherence Support for Low-Income Women With Breast Cancer														•										
2009	Griffith	Home-Based Exercise Intervention During Cancer Treatment	Not reported			•																				
2009	Irwin	Randomized controlled trial of aerobic exercise on insulin and insulin-like growth factors in breast cancer survivors: The Yale Exercise and Survivorship Study												•												

Year	First Author	Title	Source	[41]	[42]	[43]	[44]	[45]	[46]	[47]	[48]	[49]	[50]	[51]	[52]	[53]	[54]	[55]	[56]	[57]	[58]	[59]	[60]	[61]	[62]	[63]
2009	Morey	Reach Out to Enhance Wellness (RENEW) Trial	Not reported			•																				
2009	Mustian	A 4-week home-based aerobic and resistance exercise program during radiation therapy: A pilot randomized clinical trial												•												
2009	Vilhauer	Perceived benefits of online support groups for women with metastatic breast cancer	Women Health. 2009;49:381–404	•																						
2009	van Uden-Kraan	Participation in online patient support groups endorses patients' empowerment	Patient Educ Couns. 2009;74:61–69	•																						
2010	Haines	Multimodal exercise improves quality of life of women being treated for breast cancer, but at what cost? Randomized trial with economic evaluation												•												
2010	Hawkins	Comprehensive Health Enhancement Support System (CHESS) for Women With Breast Cancer														•										
2010	Høybye	Effect of Internet peer-support groups on psychosocial adjustment to cancer: a randomised study	Br J Cancer. 2010;102:1348–1354	•																						
2010	Livingston	The psychological impact of a specialist referral and telephone intervention on male cancer patients	10.1002/pon.1609		•																					
2010	Marcus	Can telephone counseling post treatment improve psychosocial outcomes among early-stage breast cancer survivors?	10.1002/pon.1653		•											•										
2010	Namkoong	Content Analysis of Treatment Information Sharing in Computer-Mediated Breast Cancer Support Groups	10.1080/10810730903528089						•																	
2010	Salzer	A randomized controlled study of Internet peer-to-peer interactions among women newly diagnosed with breast cancer	Psychooncology. 2010;19:441–446	•																						

Year	First Author	Title	Source	[41]	[42]	[43]	[44]	[45]	[46]	[47]	[48]	[49]	[50]	[51]	[52]	[53]	[54]	[55]	[56]	[57]	[58]	[59]	[60]	[61]	[62]	[63]
2011	Badger	Psychosocial interventions to improve quality of life in prostate cancer survivors and their intimate/family partners	10.1007/s11136-010-9822-2		•																					
2011	Baker	Comparing Internet-Only, CHESS Information, CHESS Information + Support, and Full CHESS for Breast Cancer Survivors														•										
2011	David	Web-Based Psychoeducational Intervention for Breast Cancer Survivors [verify]														•										
2011	Djuric	Telephone-Based Weight Control Intervention During Breast Cancer Treatment	Not reported			•																				
2011	Donnelly	Home-Based Exercise Intervention for Gynecological Cancer Survivors	Not reported			•																				
2011	Hawkins	Effects of Mentorship and Support Within CHESS for Breast Cancer Patients														•										
2011	Hayes	Telephone-Delivered Physical Activity Intervention for Women Treated for Breast Cancer														•										
2011	Hoyer	A nurse-led telephone session and quality of life after radiotherapy among women with breast cancer	10.2174/1874434601105010031		•											•										
2011	Kim	Telephone Counseling for Physical Activity and Diet Among Breast Cancer Survivors	Not reported			•																				
2011	Kimman	Nurse-Led Telephone Follow-Up and an Educational Group Programme After Breast Cancer Treatment	DOI: 10.1007/s10549-011-1427-9				•									•										
2011	Klasnja	Supporting cancer patients' unanchored health information management with mobile technology	PMID: 22195130					•																		
2011	Rabin	Step Into Motion: Web-Based Physical Activity Intervention for Young Adult Cancer Survivors	Not reported			•																				

Year	First Author	Title	Source	[41]	[42]	[43]	[44]	[45]	[46]	[47]	[48]	[49]	[50]	[51]	[52]	[53]	[54]	[55]	[56]	[57]	[58]	[59]	[60]	[61]	[62]	[63]
2011	Sherman	The Effects of Psychoeducation and Telephone Counseling on the Adjustment of Women With Early-Stage Breast Cancer														•										
2012	Badger	Telephone-delivered health education and interpersonal counseling improve quality of life for Latinas with breast cancer and their supportive partners	10.1002/pon.3101		•																					
2012	Crane-Okada	Senior peer counseling by telephone for psychosocial support after breast cancer surgery	10.1188/12.ONF.78-89		•											•										
2012	Eakin	Living Well After Breast Cancer Trial	Not reported			•																				
2012	Ligibel	Randomized Trial of a Telephone-Based Physical Activity Intervention in Breast Cancer Survivors	DOI: 10.1200/JCO.2011.36.3766			•	•							•												
2012	Loiselle	Cancer Information Navigator Intervention for Newly Diagnosed Cancer Patients	DOI not reported						•																	
2012	Park	Telephone-Delivered Health Coaching by Oncology Nurses for Breast Cancer Survivors	DOI not readily identifiable				•																			
2012	Reeves	Living Well after Breast Cancer: Changes in Objectively Measured Physical Activity in a Weight Loss Trial	10.1186/1471-2407-14-780							•																
2012	Sherman	The effects of psychoeducation and telephone counseling on the adjustment of women with early-stage breast cancer	10.1016/j.apnr.2009.10.003		•																					
2013	Changrani	Online Support Group Intervention for Spanish-Speaking Immigrant Women With Breast Cancer	DOI not reported						•																	
2013	Cheville	A home-based exercise program to improve function, fatigue, and sleep quality in patients with Stage IV lung and colorectal cancer: A randomized controlled trial												•												
2013	Eakin	Living Well After Breast Cancer Trial														•										

Year	First Author	Title	Source	[41]	[42]	[43]	[44]	[45]	[46]	[47]	[48]	[49]	[50]	[51]	[52]	[53]	[54]	[55]	[56]	[57]	[58]	[59]	[60]	[61]	[62]	[63]
2013	Hatchett	E-mail Delivered Physical Activity Intervention for Breast Cancer Survivors	Not reported			•													•							•
2013	Hawkes	CanChange Trial: Multiple Health Behavior Intervention for Colorectal Cancer Survivors	Not reported			•																				
2013	Hayes	Telephone Versus Face-to-Face Exercise Intervention Following Breast Cancer Surgery	Not reported			•										•										
2013	Heo	Effects of a smartphone application on breast self-examination: a feasibility study	10.4258/hir.2013.19.4.250					•																		
2013	Kesler	Cognitive training for improving executive function in chemotherapy-treated breast cancer survivors.	https://doi.org/10.1016/j.clbc.2013.02.004.																	•						
2013	Pinto	Physical Activity Intervention for Colorectal Cancer Survivors	Not reported			•								•												
2013	Pinto	A randomized trial to promote physical activity among breast cancer patients.	Not reported			•								•		•						•				
2013	Ryhänen	Internet-Based Patient Pathway Program for Breast Cancer Patients														•										
2013	Valle	FITNET: Facebook-Based Physical Activity Intervention for Young Adult Cancer Survivors	Not reported			•																				
2013	Ziller	Adherence Support Via Telephone Follow-Up for Endocrine Therapy in Breast Cancer														•										
2014	Backman	A randomized pilot study with daily walking during adjuvant chemotherapy for patients with breast and colorectal cancer												•												
2014	Batenburg	Emotional Coping Moderates the Effects of Online Support Group Participation in Breast Cancer Patients	10.1080/08870446.2014.918972						•																	

Year	First Author	Title	Source	[41]	[42]	[43]	[44]	[45]	[46]	[47]	[48]	[49]	[50]	[51]	[52]	[53]	[54]	[55]	[56]	[57]	[58]	[59]	[60]	[61]	[62]	[63]
2014	Børøsund	Comparing effects in regular practice of e-communication and Web-based self-management support among breast cancer patients: preliminary results from a randomized controlled trial	10.2196/jmir.3348														•									
2014	Børøsund	WebChoice: A Web-Based Illness Management System for Women With Breast Cancer	10.2196/jmir.2955						•			•														
2014	Carpenter	An online stress management workbook for breast cancer.	https://doi.org/10.1007/s10865-012-9481-6.An.						•				•			•										
2014	Des Bordes	Evaluation of an Online Bone Health Educational Tool for Breast and Prostate Cancer Survivors	DOI not reported						•																	
2014	Goodwin	Lifestyle Intervention for Weight Loss Among Breast Cancer Survivors														•										
2014	Kaye	Web-Based Tailored Intervention for Physical Activity Promotion in Breast Cancer Survivors																				•				
2014	Lee	A Web-based self-management exercise and diet intervention for breast cancer survivors: pilot randomized controlled trial	10.1016/j.ijnurstu.2014.04.012														•		•							•
2014	Mayo	Pedometer-facilitated walking intervention shows promising effectiveness for reducing cancer fatigue: A pilot randomized trial												•												
2014	Min	Daily collection of self-reporting sleep disturbance data via a smartphone app in breast cancer patients receiving chemotherapy: a feasibility study	10.2196/jmir.3421					•																		
2014	Salonen	Telephone Support Intervention for Women Undergoing Breast Cancer Surgery	DOI: 10.1007/s00520-013-2041-3				•																			
2014	Weaver	A pilot study: dose adaptation of capecitabine using mobile phone toxicity monitoring – supporting patients in their homes	10.1007/s00520-014-2224-1					•																		

Year	First Author	Title	Source	[41]	[42]	[43]	[44]	[45]	[46]	[47]	[48]	[49]	[50]	[51]	[52]	[53]	[54]	[55]	[56]	[57]	[58]	[59]	[60]	[61]	[62]	[63]
2015	Demark-Wahnefried	Reach Out to Enhance Wellness (RENEW)-Related Weight Loss Intervention for Breast Cancer Survivors [verify]														●										
2015	Eden	Mammography Decision Aid Reduces Decisional Conflict for Women in Their Forties Considering Screening	10.1089/jwh.2015.5256					●																		
2015	Fazzino	A qualitative evaluation of a group phone-based weight loss intervention for rural breast cancer survivors: Themes and mechanisms of success	10.1007/s00520-016-3149-7					●																		
2015	Freeman	Telemedicine Delivery of Cognitive Behavioral Therapy for Sleep Disturbance in Breast Cancer Survivors										●				●										
2015	McCarroll	Feasibility of a lifestyle intervention for overweight/obese endometrial and breast cancer survivors using an interactive mobile application	10.1016/j.ygyno.2014.12.025					●																	●	
2015	Park	The effect of oncologists' exercise recommendations on the level of exercise and quality of life in survivors of breast and colorectal cancer: A randomized controlled trial												●												
2015	Pinto	Peers promoting physical activity among breast cancer survivors: A randomized controlled trial	https://doi.org/10.1037/hea0000120.											●								●				
2015	Rogers	Effects of the BEAT Cancer Physical Activity Behavior Change Intervention on Physical Activity, Aerobic Fitness, and Quality of Life in Breast Cancer Survivors: A Multicenter Randomized Controlled Trial	10.1007/s10549-015-3535-6							●																
2015	Wheelock	System for Individualized Survivorship Care (SIS.NET): Online Symptom Monitoring for Breast Cancer Survivors										●														

Year	First Author	Title	Source	[41]	[42]	[43]	[44]	[45]	[46]	[47]	[48]	[49]	[50]	[51]	[52]	[53]	[54]	[55]	[56]	[57]	[58]	[59]	[60]	[61]	[62]	[63]
2015	Zhu	Nurse-Led Telephone Follow-Up for Breast Cancer Patients	DOI not reported in review				•																			
2015	van den Berg	BREATH: Web-Based Self-Management for Early Breast Cancer Survivors	10.1200/JCO.2013.54.9386						•				•			•	•	•						•		
2016	Befort	Weight Loss Maintenance Intervention Delivered by Group Phone Counseling in Breast Cancer Survivors														•										
2016	Chee	Internet Cancer Support Group for Asian American Breast Cancer Survivors														•										
2016	Damholdt	Web-Based Cognitive Training (e-CogT) for Breast Cancer Survivors														•							•			
2016	Egbring	A Mobile App to Stabilize Daily Functional Activity of Breast Cancer Patients in Collaboration With the Physician: A Randomized Controlled Clinical Trial	10.2196/jmir.6414					•																		
2016	Foley	PATI: Patient accessed tailored information: A pilot study to evaluate the effect on preoperative breast cancer patients of information delivered via a mobile application	10.1016/j.breast.2016.08.012					•																		
2016	Fu	mHealth self-care interventions: managing symptoms following breast cancer treatment	10.21037/mhealth.2016.07.03														•									
2016	Galiano-Castillo	Telehealth System for Improving Quality of Life in Breast Cancer Survivors	10.1002/cncr.30172						•			•				•			•							
2016	Gokal	Effects of a self-managed home-based walking intervention on psychosocial health outcomes for breast cancer patients receiving chemotherapy: A randomised controlled trial												•												
2016	Harrigan	Telephone Versus In-Person Weight Loss Counseling for Breast Cancer Survivors														•										

Year	First Author	Title	Source	[41]	[42]	[43]	[44]	[45]	[46]	[47]	[48]	[49]	[50]	[51]	[52]	[53]	[54]	[55]	[56]	[57]	[58]	[59]	[60]	[61]	[62]	[63]
2016	Hartman	Technology- and Phone-Based Weight Loss Intervention: Pilot RCT in Women at Elevated Breast Cancer Risk	10.1016/j.amepre.2016.06.024					•																		
2016	House	A Virtual Reality-Based Upper Extremity Rehabilitation Program for Breast Cancer Survivors with Chronic Pain: A Feasibility Study	DOI not reported								•															
2016	Hu	Intensive intervention conducted via WeChat groups and WeChat official accounts																								•
2016	Hwang	Electronic wound monitoring after ambulatory breast cancer surgery: Improving patient care and satisfaction using a smart phone app	No DOI reported					•																		
2016	Jarden	Multimodal intervention integrated into the clinical management of acute leukemia improves physical function and quality of life during consolidation chemotherapy: A randomized trial "PACE-AL"												•												
2016	Kim	Depression Screening Using Daily Mental-Health Ratings from a Smartphone Application for Breast Cancer Patients	10.2196/jmir.5598					•																		
2016	Li	Microlectures and WeChat-based rehabilitation after surgery																								•
2016	Quintiliani	Mobile Health–Supported Behavioral Counseling Intervention for Breast Cancer Survivors	10.1007/s13187-016-0994-4						•																	
2016	Sajid	Novel physical activity interventions for older patients with prostate cancer on hormone therapy: A pilot randomized study												•												
2016	Uhm	Effects of Exercise Intervention in Breast Cancer Patients Using Mobile Application and Wearable Device	10.1007/s13187-016-1155-5					•																		

Year	First Author	Title	Source	[41]	[42]	[43]	[44]	[45]	[46]	[47]	[48]	[49]	[50]	[51]	[52]	[53]	[54]	[55]	[56]	[57]	[58]	[59]	[60]	[61]	[62]	[63]
2016	Vallance	Exploring the feasibility of a broad-reach physical activity behavior change intervention for women receiving chemotherapy for breast cancer: A randomized trial												•								•				
2016	Valle	Preventing Weight Gain in African American Breast Cancer Survivors Using Smart Scales and Activity Trackers: A Randomized Controlled Pilot Study	10.1007/s13187-016-1132-z					•		•																
2017	Abrahams	The efficacy of internet-based based cognitive behavioral therapy for severely fatigued survivors of breast cancer compared with care as usual: a randomized controlled trial.	https://doi.org/10.1002/cncr.30815.										•			•		•						•		
2017	Admiraal	ENCOURAGE: Web-Based Psychoeducational Intervention for Breast Cancer Patients	https://doi.org/10.1016/j.jpainsymman.2017.1007.1009.									•						•								
2017	Alanzi	Evaluation of the SnapChat mobile social networking application for breast cancer awareness among Saudi students	10.2147/BCTT.S166135					•																		
2017	Bruggeman	Web-Based Mindfulness-Based Cognitive Therapy for Breast Cancer Survivors														•										
2017	Buche	Virtual Reality for Anxiety and Emotional Management in Breast Cancer Rehabilitation: A Pilot Study	DOI not reported								•															
2017	Chee	Internet Cancer Support Group for Asian American Breast Cancer Survivors	DOI not reported						•																	
2017	Cox	Online Lifestyle Intervention for Breast Cancer Survivors														•										
2017	Fazzino	Telephone-Based Weight Loss Program for Breast Cancer Survivors										•														
2017	Galiano-Castillo	Effect of an Internet-based telehealth system on functional capacity and cognition in breast cancer survivors: a secondary analysis of a randomized controlled trial	10.1007/s00520-017-3782-9																•							

Year	First Author	Title	Source	[41]	[42]	[43]	[44]	[45]	[46]	[47]	[48]	[49]	[50]	[51]	[52]	[53]	[54]	[55]	[56]	[57]	[58]	[59]	[60]	[61]	[62]	[63]
2017	Gordon	Cost-Effectiveness of Telephone-Delivered Exercise Intervention in Breast Cancer Survivors														•										
2017	Han	Comprehensive Health Enhancement Support System (CHESS) for Breast Cancer Patients										•														
2017	Han	Breast and cervical cancer screening literacy among Korean American women: A community health worker–led intervention.														•										
2017	Harder	A user-centred approach to developing bWell, a mobile app for arm and shoulder exercises after breast cancer treatment	10.1007/s11764-017-0630-3					•																		
2017	Hawkins	Effects of Comprehensive Health Enhancement Support System (CHESS) With Mentor Support	DOI not reported						•																	
2017	Hummel	Internet-Based Cognitive Behavioral Therapy for Sexual Dysfunction in Breast Cancer Survivors	10.1200/JCO.2016.69.6021						•				•					•					•			
2017	Keohane	Can an educational application increase risk perception accuracy amongst patients attending a high-risk breast cancer clinic?	10.1016/j.breast.2017.02.009					•																		
2017	Kim	Depression and eHealth Interventions in Breast Cancer Patients: Effects of CHESS and Mentor Support	DOI not reported						•																	
2017	Lee	Mobile Phone Multilevel and Multimedia Messaging Intervention for Breast Cancer Screening: Pilot Randomized Controlled Trial	10.2196/mhealth.7091					•																		
2017	Mendoza	A Fitbit and Facebook mHealth intervention for promoting physical activity among adolescent and young adult childhood cancer survivors: A pilot study												•												

Year	First Author	Title	Source	[41]	[42]	[43]	[44]	[45]	[46]	[47]	[48]	[49]	[50]	[51]	[52]	[53]	[54]	[55]	[56]	[57]	[58]	[59]	[60]	[61]	[62]	[63]
2017	Nguyen	A Qualitative Evaluation of Breast Cancer Survivors' Acceptance of and Preferences for Consumer Wearable Technology Activity Trackers	10.2196/mhealth.7048							•																
2017	Pinto	Does a peer-led exercise intervention affect sedentary behavior among breast cancer survivors?	https://doi.org/10.1002/pon.4255.																			•				
2017	Reeves	Living Well After Breast Cancer																				•				
2017	Shaw	Insightful Disclosure Within Computer-Mediated Support Groups and Breast Cancer Outcomes	10.1080/10810730.2016.1250843						•																	
2017	Uhm	Effects of Exercise Intervention in Breast Cancer Patients: Is Mobile Health (mHealth) with Pedometer More Effective than Conventional Program Using Brochure?	10.1007/s13187-016-1155-5						•			•		•							•					
2017	Valle	Preventing Weight Gain in African American Breast Cancer Survivors Using Smart Scales and Activity Trackers																				•				
2017	Wallwiener	Reliability of an e-PRO Tool of EORTC QLQ-C30 for Measurement of Health-Related Quality of Life in Patients With Breast Cancer	10.2196/jmir.8210					•																		
2018	Ainsworth	Acceptability of a Mobile Phone App for Measuring Time Use in Breast Cancer Survivors	DOI not reported in review					•																		
2018	Atema	Internet-Based Cognitive Behavioral Therapy for Treatment-Induced Menopausal Symptoms in Breast Cancer Survivors	10.1200/JCO.2018.78.8567															•								
2018	Buscemi	My Guide: A Mobile Phone–Based Intervention to Improve Quality of Life in Hispanic Breast Cancer Survivors	DOI not reported in review					•																		
2018	Chapman	Pilot randomized trial of a volitional help sheet-based tool to increase leisure time physical activity in breast cancer survivors.	https://doi.org/10.1111/bjhp.12313.																	•						•

Year	First Author	Title	Source	[41]	[42]	[43]	[44]	[45]	[46]	[47]	[48]	[49]	[50]	[51]	[52]	[53]	[54]	[55]	[56]	[57]	[58]	[59]	[60]	[61]	[62]	[63]
2018	Cheung	Lessons in Linking Affect and Coping (LILAC): Online Positive Affect Skills Intervention	10.1007/s00520-018-4302-z						•																	
2018	Ferrante	Technology-Supported Weight Management in Older African American Breast Cancer Survivors														•										
2018	Golsteijn	Short-term efficacy of a computer-tailored physical activity intervention for prostate and colorectal cancer patients and survivors: A randomized controlled trial												•												
2018	Graetz	Use of a web-based app to improve breast cancer symptom management and adherence for aromatase inhibitors: A randomized controlled feasibility trial	10.1007/s11764-018-0682-z												•						•					
2018	Hartman	Patterns of Fitbit Use and Activity Levels Throughout a Physical Activity Intervention: Exploratory Analysis from a Randomized Controlled Trial	10.2196/mhealth.10040							•						•										
2018	Hartman	Randomized controlled trial of increasing physical activity on objectively measured and self-reported cognitive functioning among breast cancer survivors:								•				•								•				
2018	Hawkins	Combined CHESS and Cancer Information Mentor Intervention for Women With Breast Cancer	DOI not reported						•																	
2018	Jin	Application of Virtual Reality Rehabilitation Training System in Functional Recovery after Breast Cancer Surgery	DOI not reported								•															
2018	Kim	A Mobile Game for Patients with Breast Cancer for Chemotherapy Self-Management and Quality-of-Life Improvement: Randomized Controlled Trial	10.2196/jmir.9559									•			•	•	•		•		•					

Year	First Author	Title	Source	[41]	[42]	[43]	[44]	[45]	[46]	[47]	[48]	[49]	[50]	[51]	[52]	[53]	[54]	[55]	[56]	[57]	[58]	[59]	[60]	[61]	[62]	[63]
2018	Kleckner	Effects of exercise during chemotherapy on chemotherapy-induced peripheral neuropathy: A multicenter randomized controlled trial												●												
2018	Lee	Development and Evaluation of Culturally and Linguistically Tailored Mobile App to Promote Breast Cancer Screening	10.3390/jcm7080181					●																		
2018	Lengacher	Mobile Mindfulness-Based Stress Reduction for Breast Cancer (mMBSR(BC))	10.1002/pon.4984					●	●																	
2018	Lozano-Lozano	BENECA mHealth System for Monitoring Energy Balance in Breast Cancer Survivors	DOI not reported in review					●																		
2018	Lynch	Study Design and Methods for the ACTIVity And TEchnology (ACTIVATE) Trial	10.1016/j.cct.2017.11.001							●																
2018	McCarthy	Feasibility of a Telemedicine-Delivered Cognitive Behavioral Therapy for Insomnia in Rural Breast Cancer Survivors										●														
2018	Meneses	Early Education and Support Intervention for Breast Cancer Survivors														●										
2018	Orlemann	Use of a mobile application to support nutrition therapy in patients undergoing oncological treatment: a randomized controlled pilot trial	10.1016/j.clnu.2017.07.015																						●	
2018	Pope	Effectiveness of Combined Smartwatch and Social Media Intervention on Breast Cancer Survivor Health Outcomes: A 10-Week Pilot Randomized Trial	10.3390/jcm7060140							●				●								●				●
2018	Rosen	Quality of life among women diagnosed with breast cancer: A randomized waitlist controlled trial of commercially available mobile app-delivered mindfulness training	10.1002/pon.4764					●																●		
2018	Sherman	Web-Based Body Image Intervention for Breast Cancer Survivors														●										

Year	First Author	Title	Source	[41]	[42]	[43]	[44]	[45]	[46]	[47]	[48]	[49]	[50]	[51]	[52]	[53]	[54]	[55]	[56]	[57]	[58]	[59]	[60]	[61]	[62]	[63]
2018	Stubbins	FitSurvivor: a pilot study of an mHealth obesity intervention for breast cancer survivors	10.1186/s12885-018-4302-0																						•	
2018	Urech	STREAM: Therapist-Guided Web-Based Stress Management for Cancer Patients	10.2196/jmir.10685						•																	
2018	Visser	My-GMC: Group Medical Consultations Combined with an Online Application for Breast Cancer Survivors	DOI not reported in review					•				•									•					
2018	Wang	QQ platform–based continuing nursing intervention																								•
2018	Zachariae	Internet-Delivered Cognitive-Behavioral Therapy for Insomnia in Breast Cancer Survivors: A Randomized Controlled Trial										•	•			•										
2018	Zhu	Mobile Breast Cancer e-Support Program for Chinese Women With Breast Cancer Undergoing Chemotherapy (Part 1): Qualitative Study	10.2196/mhealth.9311					•							•											
2018	Zhu	Mobile Breast Cancer e-Support Program for Chinese Women With Breast Cancer Undergoing Chemotherapy (Part 2): Multicenter Randomized Controlled Trial	10.2196/mhealth.9438					•													•					
2019	Ariza-Garcia	A Web-Based Exercise System (e-CuidateChemo) to Counter the Side Effects of Chemotherapy in Patients with Breast Cancer: Randomized Controlled Trial										•				•			•							•
2019	Atema	Efficacy of internet-based cognitive behavioral therapy for treatment-induced menopausal symptoms in breast cancer survivors: results of a randomized controlled trial																					•			
2019	Bani Mohammad	Virtual Reality-Based Intervention for Pain and Anxiety Management in Women with Breast Cancer	DOI not reported								•															

Year	First Author	Title	Source	[41]	[42]	[43]	[44]	[45]	[46]	[47]	[48]	[49]	[50]	[51]	[52]	[53]	[54]	[55]	[56]	[57]	[58]	[59]	[60]	[61]	[62]	[63]
2019	Buscemi	My Guide and My Health: mHealth intervention for Latina breast cancer survivors	10.1007/s13187-018-1376-4																						•	
2019	Cadmus-Bertram	Building a physical activity intervention into clinical care for breast and colorectal cancer survivors in Wisconsin: A randomized controlled pilot trial												•												
2019	Chen	Effects of Virtual Cognitive Training on Cognitive Impairment after Chemotherapy in Breast Cancer Patients	DOI not reported								•															
2019	Cheville	Effect of collaborative telerehabilitation on functional impairment and pain among patients with advanced-stage cancer: A randomized clinical trial												•												
2019	Dong	Combined Exercise Intervention Based on Internet and Social Media Software for Breast Cancer Survivors																	•							
2019	Hartman	Mediators of a Physical Activity Intervention on Cognition in Breast Cancer Survivors: Evidence from a Randomized Controlled Trial	10.2196/13150							•																
2019	Im	Technology-Based Information and Support Intervention for Breast Cancer Survivors														•										
2019	Imai	Problem-Solving Therapy Mobile App for Fear of Cancer Recurrence																			•					
2019	Kenfield	Feasibility, acceptability, and behavioral outcomes from a technology-enhanced behavioral change intervention (Prostate 8): A pilot randomized controlled trial in men with prostate cancer												•												
2019	Kim	Effects of a 12-week home-based exercise program on quality of life, psychological health, and physical activity in colorectal cancer survivors: A randomized controlled trial												•												

Year	First Author	Title	Source	[41]	[42]	[43]	[44]	[45]	[46]	[47]	[48]	[49]	[50]	[51]	[52]	[53]	[54]	[55]	[56]	[57]	[58]	[59]	[60]	[61]	[62]	[63]
2019	Krok-Schoen	Mobile Medication Adherence App with SMS Reminders for Breast Cancer Survivors																			•					
2019	Lee	Smartphone application vs. pedometer to promote physical activity in prostate cancer patients												•												
2019	Lynch	A Randomized Controlled Trial of Wearable Technology-Based Intervention for Increasing Moderate-to-Vigorous Physical Activity and Reducing Sedentary Behavior in Breast Cancer Survivors: The ACTIVATE Trial	10.1007/s10549-019-05338-9							•				•		•			•			•				•
2019	Lynch	Maintenance of Physical Activity and Sedentary Behavior Change, and Physical Activity and Sedentary Behavior Change After an Abridged Intervention: Secondary Outcomes from the ACTIVATE Trial	10.1186/s12885-019-5980-3							•																
2019	Maxwell-Smith	A randomized controlled trial of WATAAP to promote physical activity in colorectal and endometrial cancer survivors												•												
2019	McNeil	Activity Tracker to Prescribe Various Exercise Intensities in Breast Cancer Survivors	10.1249/MSS.0000000000001890.							•				•					•			•				
2019	Nemli	Home-Based Exercise Training with Telephone Follow-Up for Breast Cancer Survivors																	•							
2019	Nápoles	trackC Mobile Survivorship Support Program																			•					
2019	Paladino	Mobile App–Based Adherence and Symptom Management During Endocrine Therapy														•										
2019	Park	The effects of utilizing smartphone application peer support on health behavior and body mass index among breast cancer survivors.	https://doi.org/10.4040/jkan.2019.49.5.550.																•	•						

Year	First Author	Title	Source	[41]	[42]	[43]	[44]	[45]	[46]	[47]	[48]	[49]	[50]	[51]	[52]	[53]	[54]	[55]	[56]	[57]	[58]	[59]	[60]	[61]	[62]	[63]
2019	Pope	Combined Mobile App and Facebook Physical Activity Intervention for Breast Cancer Survivors	10.1093/tbm/iby002					●																		
2019	Smith	Reimagine: a randomized controlled trial of an online, symptom self-management curriculum among breast cancer survivors	10.1007/s00520-018-4431-7														●						●			
2019	Van Blarigan	Self-monitoring and reminder text messages to increase physical activity in colorectal cancer survivors (Smart Pace): A pilot randomized controlled trial												●												
2019	Weiner	Breast Cancer Survivors Reduce Accelerometer-Measured Sedentary Time in an Exercise Intervention	10.1007/s10549-019-05197-4							●												●				
2019	Zhu	Virtual Reality Rehabilitation Training for Upper Limb Function Recovery after Breast Cancer Surgery	DOI not reported								●															●
2019	van Helmondt	eHealth-Supported Cognitive Behavioral Therapy for Fear of Cancer Recurrence in Breast Cancer Survivors	10.1002/pon.4973															●								
2020	Atef	Effect of Virtual Reality Exercises on Breast Cancer-Related Lymphedema	DOI not reported								●															
2020	Baik	Social media–based intervention among Latina breast cancer survivors: pilot randomized controlled trial	10.2196/15543																						●	
2020	Bellens	A videogame-based cognitive training for breast cancer survivors with cognitive impairment: a prospective randomized pilot trial																					●			
2020	Cairo	Evaluation of a mobile health intervention to improve wellness outcomes for breast cancer survivors	10.17294/2330-0698.1733												●										●	
2020	Chirico	Virtual Reality in Health System: Beyond Entertainment. A Mini-Review on the Efficacy of VR During Cancer Treatment (Breast cancer subgroup trial)	10.1007/s00520-019-05131-8								●															

Year	First Author	Title	Source	[41]	[42]	[43]	[44]	[45]	[46]	[47]	[48]	[49]	[50]	[51]	[52]	[53]	[54]	[55]	[56]	[57]	[58]	[59]	[60]	[61]	[62]	[63]
2020	Chow	HealthWatch: a social and behavioural mHealth intervention for adolescent and young adult cancer survivors	10.2196/15438																						•	
2020	Chung	Exercise promotion and distress reduction using a mobile app-based community in breast cancer survivors	10.3389/fonc.2019.01505												•											
2020	Crafoord	Engagement in an Interactive App for Symptom Self-Management During Treatment in Patients with Breast or Prostate Cancer: Mixed Methods Study										•														
2020	Ferrante	mHealth and eHealth Weight-Loss Intervention in Older African American Breast Cancer Survivors										•														
2020	Feyzioğlu	The Effectiveness of Xbox Kinect-Based Virtual Reality Training on Upper Extremity Function in Breast Cancer Survivors	10.1007/s00520-020-05521-6								•															•
2020	Fjell	Reduced symptom burden with the support of an interactive app during neoadjuvant chemotherapy for breast cancer – a randomized controlled trial	10.1016/j.breast.2020.03.004									•			•						•					
2020	Fuemmeler	Mobile game intervention for adolescent and young adult cancer survivors	10.1002/pon.5487																						•	
2020	Grašič Kuhar	Mobile app for symptom management and associated quality of life during systemic treatment in early-stage breast cancer: Nonrandomized controlled prospective cohort study	10.2196/17408												•						•					
2020	Hou	Quality of life of women after a first diagnosis of breast cancer using a self-management support mHealth app in Taiwan: Randomized controlled trial	10.2196/17084									•			•	•	•		•		•					
2020	Lally	CaringGuidance: Web-Based Psychoeducational Distress Self-Management Program										•														

Year	First Author	Title	Source	[41]	[42]	[43]	[44]	[45]	[46]	[47]	[48]	[49]	[50]	[51]	[52]	[53]	[54]	[55]	[56]	[57]	[58]	[59]	[60]	[61]	[62]	[63]
2020	Lleras de Frutos	Positive Psychology Intervention Delivered Through Videoconferencing for Breast Cancer Survivors														•										
2020	Lozano-Lozano	Mobile health and supervised rehabilitation versus mobile health alone in breast cancer survivors: Randomized controlled trial	10.1016/j.rehab.2019.07.007									•			•						•					
2020	Marinac	Effects of a Remote Lifestyle Intervention on Sedentary Behavior and Physical Activity in Older Breast Cancer Survivors	10.1007/s10549-020-05534-2							•																
2020	Meneses	Rural Breast Cancer Survivorship Education and Support Program														•										
2020	Møller	Instructed Pedometer Intervention																				•				
2020	Pinto	SMS text message intervention																								•
2020	Santa-Maria	POWER-Remote: Telephone-Based Behavioral Coaching and Web-Based Self-Monitoring Platform																	•							
2020	Singh	Feasibility and effect of a physical activity counselling session with or without provision of an activity tracker on maintenance of physical activity in women with breast cancer—A randomised controlled trial												•								•				•
2020	Tang	Standardized video education and WeChat group rehabilitation																								•
2020	Vallance	ACTIVATE Trial Quality-of-Life Outcomes Using a Wrist-Worn Activity Tracker																	•							
2020	Yanez	MyGuide Mobile App for Latina Breast Cancer Survivors																			•					
2020	van der Hout	Role of eHealth Application Oncokompas in Supporting Self-Management of Symptoms and Health-Related Quality of Life in Cancer Survivors: A Randomized Controlled Trial										•														

Year	First Author	Title	Source	[41]	[42]	[43]	[44]	[45]	[46]	[47]	[48]	[49]	[50]	[51]	[52]	[53]	[54]	[55]	[56]	[57]	[58]	[59]	[60]	[61]	[62]	[63]
2021	Allicock	Creating Healthy Actions Through Technology (CHAT): a pilot randomized controlled trial among African American breast cancer survivors	10.1007/s13187-020-01880-4																						•	
2021	Dong	Remote-guided exercise intervention with exercise videos and social media support																								•
2021	Fang	Long-Term Effectiveness of a Decision Support App (Pink Journey) for Women Considering Breast Reconstruction Surgery: Pilot Randomized Controlled Trial										•														
2021	Ghanbari	Effects of psychoeducational interventions using mobile apps and mobile-based online group discussions on anxiety and self-esteem in women with breast cancer: Randomized controlled trial	10.2196/19262												•						•					
2021	Gong	Effect of computerized cognitive behavioral therapy on anxiety, depression and sleep quality of perioperative breast cancer patients	10.12102/j.issn.1009-6493.2021.23.032																					•		
2021	Holtdirk	Optimune: Web-Based Cognitive Behavioural Intervention for Breast Cancer Survivors	10.2196/26948															•	•				•	•		
2021	Jimenez	Virtual Environment for Radiotherapy Training Reduces Anxiety and Improves Knowledge in Breast Cancer Patients									•															
2021	Keum	Mobile app–based nutritional intervention in pancreatic cancer patients receiving chemotherapy	10.3390/nu13062089																						•	
2021	Krzyzanowska	Remote, Proactive, Telephone-Based Management of Toxicity in Outpatients During Adjuvant or Neoadjuvant Chemotherapy for Early Stage Breast Cancer: Pragmatic Cluster Randomised Trial										•														

Year	First Author	Title	Source	[41]	[42]	[43]	[44]	[45]	[46]	[47]	[48]	[49]	[50]	[51]	[52]	[53]	[54]	[55]	[56]	[57]	[58]	[59]	[60]	[61]	[62]	[63]
2021	Kumar	Technology Connects Patients to Tertiary Care for Non-COVID Illnesses in Pandemic Times: A Case Study from India										•														
2021	Lai	Feasibility of Remote Occupational Therapy Services via Telemedicine in a Breast Cancer Recovery Program										•														
2021	Lu	Video-based postoperative rehabilitation education																								•
2021	Oswald	Effects of Smartphone Interventions on Cancer Knowledge and Coping Among Latina Breast Cancer Survivors	10.1007/s13187-021-02028-8															•								
2021	Reeves	Effect of a Remotely Delivered Weight Loss Intervention in Early-Stage Breast Cancer: Randomized Controlled Trial										•							•			•				
2021	Sheean	Everyday Counts: Supervised Exercise, Telephone Coaching and Text Messaging for Metastatic Breast Cancer																	•			•				
2021	Wagner	Targeted eHealth intervention to reduce breast cancer survivors' fear of recurrence: results from the FoRtitude randomized trial	https://doi.org/10.1093/jnci/djab100.									•						•		•						
2021	Yang	Mobile application nutrition intervention during neoadjuvant chemoradiotherapy for esophageal cancer	10.3390/cancers13174318																						•	
2021	Ye	Motion capture technology–based rehabilitation program																								•
2021	Çınar	Effect of Mobile Phone App-Based Training on the Quality of Life for Women with Breast Cancer	10.1016/j.ejon.2021.101960									•						•	•		•				•	
2021	Öztürk	The Effect of the Mobile Application-Based Symptom Monitoring Process on the Symptom Control and Quality of Life in Breast Cancer Patients										•														

Year	First Author	Title	Source	[41]	[42]	[43]	[44]	[45]	[46]	[47]	[48]	[49]	[50]	[51]	[52]	[53]	[54]	[55]	[56]	[57]	[58]	[59]	[60]	[61]	[62]	[63]
2022	Akechi	Smartphone-Based Cognitive Behavioral Therapy for Fear of Cancer Recurrence Among Breast Cancer Survivors: Randomized Controlled Trial	10.2196/38628															•								
2022	Amidi	Changes in sleep following internet-delivered cognitive-behavioral therapy for insomnia in women treated for breast cancer: a 3-year follow-up assessment	10.1016/j.sleep.2022.04.020																					•		
2022	Bandani-Susan	Mobile Health Education for Fatigue Reduction and Body Image Improvement in Breast Cancer Patients										•														
2022	Basha	Xbox Kinect games for rehabilitation																								•
2022	Changizi	WhatsApp-Based Educational and Physical Activity Intervention for Breast Cancer Patients																	•							
2022	Chen	Intervention effect of networked group brief behavioral treatment on insomnia in breast cancer survivors	Thesis (Henan University)																					•		
2022	Fu	A Web- and mobile-based intervention for women treated for breast cancer to manage chronic pain and symptoms related to lymphedema: Results of a randomized clinical trial	10.2196/29485									•			•						•					
2022	Gao	Mobile Tai Chi and Health Education Program for Breast Cancer Survivors										•														
2022	Huggins	Comparing mobile app–delivered and telephone-delivered nutrition intervention in upper gastrointestinal cancer patients	10.3390/nu14112252																						•	
2022	Medina	Mobile Health Intervention for Breast Cancer Survivorship Care										•														

Year	First Author	Title	Source	[41]	[42]	[43]	[44]	[45]	[46]	[47]	[48]	[49]	[50]	[51]	[52]	[53]	[54]	[55]	[56]	[57]	[58]	[59]	[60]	[61]	[62]	[63]
2022	Mohammadzadeh	Effectiveness of a self-management mobile app on the quality of life of women with breast cancer: a study in a developing country	10.1186/s12905-022-02020-5														•									
2022	Ochi	Habit-B: Smartphone-Supported High-Intensity Interval Training Program for Breast Cancer Survivors																	•		•					
2022	Oswald	Pilot randomized controlled trial of eHealth cognitive-behavioral therapy for insomnia among Spanish-speaking breast cancer survivors										•														
2022	Park	Mobile web-based self-management program for breast cancer patients with chemotherapy-induced amenorrhoea: a quasi-experimental study	10.1002/nop2.1113														•									
2022	Peng	Effects of an online mindfulness-based intervention on fear of Cancer recurrence and quality of life among Chinese breast cancer survivors	10.1016/j.ctcp.2022.101686																					•		
2022	Pinto	SMS text message and email intervention																								•
2022	Swartz	Active video game–based physical activity support group (Pink Warrior)																								•
2022	Terranova	Long-Term Outcomes of a Remotely Delivered Weight Loss Intervention in Breast Cancer Survivors																	•							
2022	Wang	Effect of a 4-week internet-delivered mindfulness-based Cancer recovery intervention on the symptom burden and quality of life of patients with breast Cancer: randomized controlled trial	10.2196/40059																					•		
2022	Yang	Markerless motion capture technology intervention																								•

Year	First Author	Title	Source	[41]	[42]	[43]	[44]	[45]	[46]	[47]	[48]	[49]	[50]	[51]	[52]	[53]	[54]	[55]	[56]	[57]	[58]	[59]	[60]	[61]	[62]	[63]
2022	Yu	Research on the application of internet plus mindfulness based cognitive therapy in patients breast cancer recovering from	10.19793/j.cnki.1006-6411.2022.03.001																					•		
2023	Akkol-Solakoglu S	Internet-delivered cognitive behavioural therapy for depression and anxiety in breast cancer survivors: results from a randomised controlled trial	10.1002/pon.6097																				•	•		
2023	Jiang	Telephone/WeChat follow-up and WeChat official account intervention (ROM study)																								•
2023	Jung	WalkOn mobile app intervention																								•
2023	Park	Augmented reality–based digital health care system																								•
2023	Swartz	Virtual Pink Warrior intervention																								•
2023	Tawfik	A nurse versus a chatbot – the effect of an empowerment program on chemotherapy-related side effects and the self-care behaviors of women living with breast Cancer: a randomized controlled trial	10.1186/s12912-023-01243-7														•									
2023	Tian	Microvideo limb training intervention																								•
2024	Abdelmoniem Ibrahim	Pablo Handle training																								•
2024	Jiang	Telephone/WeChat follow-up and WeChat official account intervention (QoL study)																								•
2024	Li	WeChat app and smart wristband intervention																								•
2024	Luo	Kinect motion-sensing game rehabilitation prescription																								•

N = 459 study occurrences; r = 333 unique primary studies; c = 23 reviews. Covered area = N/(r × c) = 5.99%; corrected covered area (CCA) = (N − r)/(r × c − r) = 1.72% (slight overlap). Of the 333 primary studies, 261 appeared in one review, 41 in two, 17 in three, 9 in four, 1 in five and 4 in six reviews. Study details (year, first author, title and source) are reproduced as reported by the including review(s); "Not reported in review" indicates that the source was not stated in the review.

Table S5(a). Further details on intervention design and delivery characteristics of the included reviews.

Author (Year) [Ref.]	Intervention Focus	Intervention intensity/frequency (contacts per week)	Personalization / Tailoring (none / generic / individualized)	Automation level (human-assisted / hybrid / fully automated)	Human support (Y/N; type)	Platform developer (academic / commercial / hybrid)	Integration with healthcare system (standalone / EMR-linked)
Hong et al. (2011) [41]	Online interventions	Unknown	Generic	Hybrid	Y, professional support usually	Hybrid	Standalone
Okuyama et al. (2014) [42]	Telephone-delivered psychosocial counseling (CBT, interpersonal therapy, uncertainty management)	Variable, weekly to fortnightly, few monthly	Individualised	Human-assisted	Y, professional support usually	Academic	Standalone
Goode et al. (2015) [43]	Telephone counseling, web, print; behavior change programs targeting PA/diet/weight	Variable, weekly to monthly	Individualised	Human-assisted, few automated platforms/features	Y, professional support usually	Academic	Standalone
Zhang et al. (2016) [44]	Telephone-based intervention	Once per month to 6 per week	Mostly individualised, some generic	Hybrid	Y, professional support usually	Hybrid	Standalone
Jongerius et al. (2019) [45]	Mobile apps on prevention, care management, and survivorship	Variable, daily to weekly	Individualised (mostly), few generic	Hybrid, few fully automated, few human-assisted	Y, professional support usually	Some academic, some commercial	Standalone
Triberti et al. (2019) [46]	Online interventions, informational websites, workbooks, telecommunication with health providers, support groups, mobile apps.	Unknown	Mostly individualised, some generic	Hybrid	Y, professional support usually	Hybrid	Standalone
Blount et al. (2021) [47]	Wearable activity trackers (e.g., Fitbit, Garmin) often with multi-component counseling/feedback	Variable, daily to fortnightly	Individualised	Hybrid, human-assisted and wearables with automated tracking	Y, professional support usually	Commercial	Standalone
Bu et al. (2022) [48]	VR-based intervention	Unknown	Generic	Fully automated	N	Hybrid	Standalone
Kruse et al. (2022) [49]	Telehealth interventions including web, mobile, telephone and video conferences	Variable, daily to weekly	Generic (most mHealth apps), light tailoring (telemed or teleconsult)	Few fully automated, others hybrid with human support	Y, professional support usually	Academic	Standalone
Popovic et al. (2022) [50]	Online psychotherapy (CBT-based modules, stress management, insomnia programs, sexual dysfunction support)	Weekly	Individualised	Hybrid	Y, professional support usually	Academic	Standalone
Singh et al. (2022) [51]	Wearable activity trackers (e.g., Fitbit, Garmin) often with multi-component counseling/feedback	Variable, daily to weekly	Individualised	Fully automated, few hybrid with human support	Y, professional support usually	Commercial	Standalone
Wanchai et al. (2022) [52]	Web or mobile apps providing info about side effects, prevention, symptom management, cancer and treatment info	Variable, daily to weekly	Generic, light tailoring	Hybrid, some apps with human functions, otherwise fully automated	Y, some only with professional or peer support	Academic, few commercial	Standalone

Author (Year) [Ref.]	Intervention Focus	Intervention intensity/frequency (contacts per week)	Personalization / Tailoring (none / generic / individualized)	Automation level (human-assisted / hybrid / fully automated)	Human support (Y/N; type)	Platform developer (academic / commercial / hybrid)	Integration with healthcare system (standalone / EMR-linked)
Ajmera et al. (2023) [53]	Telephone based interventions, web based interventions, telemedicine, combination of internet, web, telephone and videoconferencing, wearable technology, mobile health based app	Unknown	Individualised in majority	Hybrid	Y, professional support usually	Hybrid	Standalone
An et al. (2023) [54]	Telehealth interventions including mobile and web apps and AI chatbot	Variable, daily to weekly	Individualised (mostly), few generic	Hybrid, only 1 fully automated	Y, professional support usually	Academic	Standalone
Horn et al. (2023) [55]	Mobile apps for psychological support grounded in CBT	N, no explicit mention	Generic, light tailoring (symptom-specific)	Fully automated, few human-assisted	Y, professional support usually	Academic, only 1 commercial	Standalone
Kang et al. (2023) [56]	Digital physical activity interventions to promote exercise, multi-component, various modalities	Variable, daily to weekly	Individualised (mostly), light tailoring, generic	Hybrid, few fully automated, few human-assisted	Y, professional support usually	Academic, few commercial	Standalone
Pimentel-Parra et al. (2023) [57]	Online interventions, computerised training programs, telecoaching, computers, mobile phones.	Daily	Generic	Hybrid	Y, professional support usually	Hybrid	Standalone
Saevarsdottir et al. (2023) [58]	Mobile app, message-based reminders, wearables, coaching calls, group support video calls and messages, in-person rehabilitation sessions	Unknown	Mostly individualised, some generic	Hybrid	Y, professional support usually	Hybrid	Standalone
Teo et al. (2023) [59]	Wearable technology	Unknown	Unknown	Hybrid	Y, professional support usually	Hybrid	Standalone
Zhang et al. (2024) [60]	Internet-based cognitive behavioural therapy (I-CBT) delivered via web/mobile apps	Variable, daily to weekly	Individualised	Hybrid, human-led therapy with self-guided studies	Y, but only half with professional support, self-guided interventions without	Academic	Standalone
Lin et al. (2025) [61]	Internet-based cognitive behavioural therapy (I-CBT) delivered via web platforms or mobile apps	Variable, daily to weekly	Generic, light tailoring	Fully automated, few hybrid with human support	Y, professional support usually	Academic, few commercial	Standalone
Ng et al. (2025) [62]	Commercial or cancer-specific nutrition apps; tracking, feedback, recommendations; multicomponent in some	Daily	Generic, light tailoring	Fully automated, few hybrid	N, rarely professional or peer support, intentionally without dietitians	Academic, few commercial	Standalone
Zhang et al. (2025) [63]	Social media platforms, games, applications	Unknown	Individualised in majority	Hybrid	Y, professional support usually	Hybrid	Standalone

BCT, behaviour change technique; CBT, cognitive behavioural therapy; EMR, electronic medical record; IPT, interpersonal therapy; PA, physical activity; Y, yes; N, no.

Table S5(b). Further details on the theoretical basis, behaviour change techniques, engagement and support features of the included reviews.

Author (Year) [Ref.]	Theoretical basis/BCTs	Behavior change theory used	Behavior change techniques (BCT taxonomy, up to 3)	Psychological mediators targeted (self-efficacy, motivation, etc.)	Social support elements (peer / professional / family)	Any engagement?	Engagement metrics (e.g., adherence %, average use)	Gamification / incentive features (Y/N)	Patient activation / empowerment measure
Hong et al. (2011) [41]	Behaviour change techniques	N	Self-monitoring of behaviour, goal setting, feedback on behaviour	Self-efficacy, motivation	Peer, professional	N	N	N	N
Okuyama et al. (2014) [42]	Commonly theory-guided (e.g., CBT, stress/coping)	Uncertainty in illness theory, stress and coping model	Cognitive restructuring, emotional support and active listening, psychoeducation and problem-solving	Self-efficacy, self-regulation, social support, uncertainty tolerance	Professional, peer (limited), family (limited)	N	N	N	N
Goode et al. (2015) [43]	Varied across studies; behaviour change frameworks (e.g., motivational interviewing)	Social cognitive theory (SCT) mostly	Self-monitoring of behaviour, goal setting, feedback on behaviour	Self-efficacy, motivation, self-regulation	Professional, peer (limited)	N	N	N	N
Zhang et al. (2016) [44]	CBT and IPT	CBT-based	Self-monitoring of behaviour, feedback on behaviour, psychoeducation and problem solving	Self-efficacy, self-regulation, cognitive reframing	Professional	N	N	N	N
Jongerius et al. (2019) [45]	Behavioural change techniques mentioned	N	Self-monitoring of behaviour, feedback on behaviour, psychoeducation and problem solving	Self-efficacy, uncertainty tolerance, cognitive reframing	Professional, peer	Y, compliance, module completion		N	N, except 1 with Dutch Empowerment Questionnaire
Triberti et al. (2019) [46]	Behaviour change techniques	N	Self-monitoring of behaviour, goal setting, feedback on behaviour	Self-efficacy, motivation	Peer, professional	N	N	Y	N
Blount et al. (2021) [47]	Often multi-component with feedback, self-monitoring, goal-setting	N	Self-monitoring of behaviour, goal setting, feedback on behaviour	Self-efficacy, motivation, self-regulation	Professional, peer (limited)	Y, wear time	1 study with high adherence to tracker (88%)	Y, through wearable platforms	N
Bu et al. (2022) [48]	Nil	N	Self-monitoring of behaviour, goal setting, feedback on behaviour	Self-efficacy, motivation	Professional	N	N	Y	N
Kruse et al. (2022) [49]	Most mention behavioural self-management principles	CBT-based	Self-monitoring of behaviour, behavioural activation, psychoeducation and problem solving	Self-efficacy, self-regulation, cognitive reframing	Professional, peer (limited)	Y. module completion, frequency of logging/reporting activity		N, except 1 with mobile game for chemo adherence	N
Popovic et al. (2022) [50]	CBT frameworks; skills training; self-efficacy enhancement	CBT-based	Self-monitoring of behaviour, cognitive restructuring, skill-building	Self-efficacy, self-regulation, adjustment and empowerment	Professional (limited)	Y, module completion		N	N
Singh et al. (2022) [51]	Varied, half mentioned behaviour change frameworks	N	Self-monitoring of behaviour, goal setting, feedback on behaviour	Self-efficacy, motivation, self-regulation	Professional, peer (limited)	Y, frequency of logging/reporting activity, wear time		N	N

Author (Year) [Ref.]	Theoretical basis/BCTs	Behavior change theory used	Behavior change techniques (BCT taxonomy, up to 3)	Psychological mediators targeted (self-efficacy, motivation, etc.)	Social support elements (peer / professional / family)	Any engagement?	Engagement metrics (e.g., adherence %, average use)	Gamification / incentive features (Y/N)	Patient activation / empowerment measure
Wanchai et al. (2022) [52]	Self-efficacy enhancement/self-management	N	Self-monitoring of behaviour, psychoeducation and problem-solving, feedback on behaviour	Self-efficacy, motivation, self-regulation	Professional, peer	N	N	N, except 1 with mobile game for chemo education	N
Ajmera et al. (2023) [53]	Behaviour change techniques	N	Self-monitoring of behaviour, goal setting, feedback on behaviour, cognitive reframing	Self-regulation, motivation	Professional	N	N	N	N
An et al. (2023) [54]	Behavioural change techniques mentioned	Self-management theory, CBT-based	Self-monitoring, information provision, goal setting	Self-efficacy, motivation, self-regulation	Professional, peer (limited)	N	N	N	N, except 1
Horn et al. (2023) [55]	CBT frameworks for behavioural change	CBT-based	Psychoeducation and problem solving, cognitive restructuring, behavioural activation	Self-efficacy, uncertainty tolerance, self-regulation	Professional	Y, frequency of logging/reporting activity, module completion	Reported only for very few studies	N	N, except 2 with Dutch Distress Thermometer
Kang et al. (2023) [56]	Behavioural change techniques mentioned	Social cognitive theory (SCT) mostly	Self-monitoring of behaviour, goal setting, feedback on behaviour	Self-efficacy, motivation, adjustment and empowerment	Professional, peer (limited)	Y, frequency of logging/reporting activity		N, except 1	N
Pimentel-Parra et al. (2023) [57]	Cognitive behavioural therapy	CBT-based	Problem solving, instruction on how to perform a behaviour, social support	Self-efficacy, cognitive appraisal, motivation	Peer, professional	N	N	Y	N
Saevarsdottir et al. (2023) [58]	Behaviour change techniques	CBT-based	Self-monitoring of behaviour, feedback on behaviour, psychoeducation and problem solving	Self-efficacy, cognitive appraisal, motivation	Professional	N	N	N	N
Teo et al. (2023) [59]	Behavioural change techniques	CBT-based	Self-monitoring of behaviour, goal setting, feedback on behaviour	Self-efficacy, motivation	Peer, professional	N	N	N	N
Zhang et al. (2024) [60]	CBT frameworks for behavioural change	CBT-based	Self-monitoring of behaviour, cognitive restructuring, behavioural activation	Self-efficacy, self-regulation	Professional	Y, module completion		N, except 1 with CBT videogame	N
Lin et al. (2025) [61]	CBT frameworks for behavioural change	CBT-based	Cognitive restructuring, behavioural activation, relaxation and mindfulness	Self-efficacy, self-regulation, mindfulness, cognitive reframing	Professional	Y, module completion		N	N, except 1 with Cancer Empowerment Questionnaire
Ng et al. (2025) [62]	Not consistently reported across studies	N	Self-monitoring of behaviour, goal setting, information provision	Self-efficacy, motivation, knowledge and awareness	Peer (limited)	Y, frequency of logging/reporting activity		N	N

Author (Year) [Ref.]	Theoretical basis/BCTs	Behavior change theory used	Behavior change techniques (BCT taxonomy, up to 3)	Psychological mediators targeted (self-efficacy, motivation, etc.)	Social support elements (peer / professional / family)	Any engagement?	Engagement metrics (e.g., adherence %, average use)	Gamification / incentive features (Y/N)	Patient activation / empowerment measure
Zhang et al. (2025) [63]	Behaviour change techniques	CBT-based	Social support, instruction on how to perform the behaviour, demonstration of the behaviour.	Self-efficacy, motivation, reflection.	Professional	N	N	Y	N

Behaviour change techniques follow the BCT taxonomy (up to three per review). CBT, cognitive behavioural therapy; SCT, social cognitive theory; Y, yes; N, no.

Table S5(c). Further details on implementation, equity, follow-up and quality characteristics of the included reviews.

Author (Year) [Ref.]	Risk of bias / quality notes	Digital literacy requirement (low / moderate / high)	Cost to user (free / paid / not stated)	Data privacy / ethics statements present (Y/N)	Adoption or scalability noted (Y/N; description)	Maintenance / follow-up evidence (Y/N)	Equity considerations (older, rural, low SES participants)	Attrition rate (%)	Follow-up duration (months)	Economic or resource-use data (Y/N)	Additional outcomes beyond QoL/PA (fatigue, sleep, cognitive)
Hong et al. (2011) [41]	Low overall bias	Moderate	Not stated	Y	N	N	N	N	N	N	Psychosocial wellbeing
Okuyama et al. (2014) [42]	CONSORT-based appraisal; limitations include heterogeneity and reporting quality	Low	Not stated	Y	Y, convenient, exportable, and low barrier	Y, but only few did; 4 with significant effects and 1 with sustained effects	N, only few with Latinas and African-Americans	Variable, 0 to 32	3 to 12	N	Stress, social support, psychosocial outcomes, uncertainty tolerance, personal growth
Goode et al. (2015) [43]	Heterogeneous; more research needed on SMS/apps; generalizability and long-term outcomes limited	Low to moderate	Not stated	Y	Y, though more research on integration with systems needed	Y, but only few did and effect sizes were small	N	Variable, 5 to 45	3 to 12	N	Fatigue, physical function, cardiorespiratory fitness, body composition, mood, pain, sleep quality, biomarkers
Zhang et al. (2016) [44]	Low overall bias	Low	Not stated	Y	N	N	N	N	N	N	Social-domestic function, self-efficiency, mood
Jongerius et al. (2019) [45]	Heterogeneous, half strong but half moderate-to-weak quality	Low to high, some only simple tracking, others require app navigation and multimedia content	Not stated	Y	Y, accessible and scalable but without real-world data	Y, but only few did	Y, some rural, minorities (Latinas, Asian-Americans, African-Americans)	N	1 to 6	N, except 1 wound-monitoring app reduced unscheduled visits and readmission (implied resource-use benefit)	Fatigue, sleep quality, cognitive function, side effects and symptoms, adherence, readmission
Triberti et al. (2019) [46]	Low overall bias	Low to moderate	Not stated	Y	Y, scalable to include broader time range and broader eHealth implementations	N	N	N	N	N	Health literacy, physical exercise
Blount et al. (2021) [47]	Varied; multi-component designs yielded greater effects	Low to moderate	Not stated	Y	N, but limited real-world implementation evidence noted	Y	N	Variable, 10 to 25	3 to 6	N	Fatigue, cardiorespiratory fitness, body composition, sleep quality, psychosocial outcomes

Author (Year) [Ref.]	Risk of bias / quality notes	Digital literacy requirement (low / moderate / high)	Cost to user (free / paid / not stated)	Data privacy / ethics statements present (Y/N)	Adoption or scalability noted (Y/N; description)	Maintenance / follow-up evidence (Y/N)	Equity considerations (older, rural, low SES participants)	Attrition rate (%)	Follow-up duration (months)	Economic or resource-use data (Y/N)	Additional outcomes beyond QoL/PA (fatigue, sleep, cognitive)
Bu et al. (2022) [48]	4 moderate risk, 2 critical risk.	Low	Not stated	Y	N	N	N	N	N	N	Incidence of complications, fatigue, anxiety. depression, fear of movement.
Kruse et al. (2022) [49]	Most are high quality, some exhibit selection bias and limited blinding	Low to moderate	Not stated	Y	Y, accessible and scalable but without real-world data	Y, but only few did	N	Variable, not consistently reported, 10 to 30	1 to 6	N	Sleep quality, pain, mood, side effects and symptoms, body composition
Popovic et al. (2022) [50]	Small number of RCTs; logistical/privacy limitations noted	Moderate	Not stated	Y	N, but limited real-world implementation evidence noted	N	N	Variable, 3 to 24	1 to 3	N	Fatigue, sleep quality, sexual function, stress, psychosocial outcomes
Singh et al. (2022) [51]	Moderate quality	Low to moderate	Not stated	Y	N, but limited real-world implementation evidence noted	N	N	N	N	N	Fatigue, cardiorespiratory fitness
Wanchai et al. (2022) [52]	Most studies low risk, no meta-analysis due to heterogeneity	Low to moderate	Not stated	Y	Y, mobile apps easily scalable but with limitations such as stable internet	N	N	N	1 to 6	N	Side effects and symptoms, emotional function, adherence
Ajmera et al. (2023) [53]	Moderate	Moderate	Not stated	Y	N	N	N	N	4 to 12	N	BMI, dietary intake, emotional processing.
An et al. (2023) [54]	Moderate quality	Low to moderate	Not stated	Y	Y, viewed as feasible and scalable, depending on digital literacy and integration	Y, but only few did	N, but some were studies from LMICs	N	2 to 6	N	Menopausal symptoms, fatigue, pain, adherence, psychosocial outcomes, dietary quality
Horn et al. (2023) [55]	Moderate quality	Moderate	Not stated	Y	Y, challenges with engagement, need for standardised development guidelines	Y, but only few did	N, only few with Latinas	Variable, not consistently reported, 3 to 36	1 to 10	N	Fatigue, menopausal symptoms, sleep quality, sexual function, physical function
Kang et al. (2023) [56]	Mostly low-to-moderate risk, limited blinding in some	Low to moderate	Not stated	Y	Y, supported by high retention, but lack of long-term trials	Y, but only few did	N	Variable, 0 to 32	6 to 18	N	Functional capacity, body composition, fatigue, side effects and symptoms, psychosocial outcomes
Pimentel-Parra et al. (2023) [57]	Low overall bias	Moderate	Not stated	Y	N, difficulty with small number of studies in specific patient subset	N	N	N	1	N	Physical, psychological and social domains
Saevarsdottir et al. (2023) [58]	Moderate	Low	Not stated	Y	N	N	N	N	N	N	Fear of recurrence, physical activity, mood
Teo et al. (2023) [59]	Low overall bias	Low	Not stated	Y	Y	N	N	2.3 to 33.3	6 to 8	N	N

Author (Year) [Ref.]	Risk of bias / quality notes	Digital literacy requirement (low / moderate / high)	Cost to user (free / paid / not stated)	Data privacy / ethics statements present (Y/N)	Adoption or scalability noted (Y/N; description)	Maintenance / follow-up evidence (Y/N)	Equity considerations (older, rural, low SES participants)	Attrition rate (%)	Follow-up duration (months)	Economic or resource-use data (Y/N)	Additional outcomes beyond QoL/PA (fatigue, sleep, cognitive)
Zhang et al. (2024) [60]	Inadequate blinding in 5 of 7, 1 high-risk, generally homogeneous otherwise	Moderate	Not stated	Y	Y, suitability for survivors returning to work but without real-world data	Y, but only few did and effect sizes were small	N	Variable, 7 to 30	3 to 6	N	Sexual function, sleep quality, cognitive function
Lin et al. (2025) [61]	Moderate quality	Low to moderate	Not stated	Y	Y, accessible and scalable but without real-world data	Y, but only few did	N	Variable, 5 to 30	N	N	Fatigue, sleep quality, side effects and symptoms, uncertainty tolerance, mindfulness, stress
Ng et al. (2025) [62]	Mostly low-to-moderate quality; small samples; heterogeneity; need RCTs	Moderate	Not stated	Y	Y, scalability limited by low tailoring and inconsistent reporting	Y, but only few did and effect sizes were small	N, only 1 incl. Latinas	Variable, 0 to 50	3 to 12	N	Sleep quality, body composition, side effects and symptoms
Zhang et al. (2025) [63]	Low overall bias	Low	Not stated	Y	N	N	N	N	3 to 6	N	Shoulder ROM, finger climbing wall height, upper-extremity function

Attrition rate and follow-up duration are reported as ranges across the primary studies of each review where available. LMIC, low- and middle-income country; SES, socioeconomic status; Y, yes; N, no.

Table S6. Reporting of determinants of real-world effectiveness across the 23 included reviews (data underlying Figure 3 of the main manuscript).

Author (Year)	Engagement or Adherence	Attrition	Long-Term Follow-Up	System Integration	Scalability	Economic Data	Equity Considerations
Hong et al. (2011) [41]	Partial	Partial	Reported	Absent	Partial	Absent	Reported
Okuyama et al. (2014) [42]	Partial	Reported	Reported	Partial	Partial	Partial	Reported
Goode et al. (2015) [43]	Reported	Reported	Reported	Partial	Reported	Partial	Reported
Zhang et al. (2016) [44]	Partial	Partial	Partial	Absent	Partial	Absent	Absent
Jongerius et al. (2019) [45]	Reported	Reported	Reported	Partial	Partial	Partial	Partial
Triberti et al. (2019) [46]	Reported	Partial	Partial	Reported	Reported	Partial	Partial
Blount et al. (2021) [47]	Reported	Reported	Reported	Partial	Reported	Partial	Reported
Bu et al. (2022) [48]	Reported	Reported	Reported	Partial	Partial	Absent	Absent
Kruse et al. (2022) [49]	Reported	Partial	Partial	Reported	Partial	Reported	Partial
Popovic et al. (2022) [50]	Partial	Partial	Reported	Partial	Partial	Absent	Reported
Singh et al. (2022) [51]	Reported	Partial	Reported	Reported	Partial	Partial	Partial
Wanchai et al. (2022) [52]	Reported	Absent	Partial	Reported	Partial	Absent	Partial
Ajmera et al. (2023) [53]	Reported	Partial	Reported	Partial	Reported	Reported	Reported
An et al. (2023) [54]	Reported	Partial	Partial	Partial	Reported	Absent	Partial
Horn et al. (2023) [55]	Reported	Reported	Reported	Partial	Partial	Absent	Partial
Kang et al. (2023) [56]	Reported	Reported	Reported	Partial	Partial	Partial	Partial
Pimentel-Parra et al. (2023) [57]	Reported	Reported	Reported	Reported	Reported	Reported	Reported
Saevarsdottir et al. (2023) [58]	Reported	Partial	Reported	Reported	Reported	Partial	Partial
Teo et al. (2023) [59]	Reported	Reported	Reported	Reported	Reported	Partial	Partial
Zhang et al. (2024) [60]	Reported	Reported	Reported	Reported	Reported	Partial	Absent
Lin et al. (2025) [61]	Reported	Partial	Partial	Reported	Reported	Partial	Partial
Ng et al. (2025) [62]	Reported	Reported	Reported	Reported	Reported	Partial	Reported
Zhang et al. (2025) [63]	Reported	Reported	Reported	Partial	Reported	Partial	Partial
**Reported, n (%)**	**19 (83%)**	**12 (52%)**	**17 (74%)**	**10 (43%)**	**12 (52%)**	**3 (13%)**	**8 (35%)**

Reported: data reported or discussed extensively; Partial: partially reported or mentioned briefly; Absent: not mentioned. System integration refers to linkage with electronic medical records or embedding within care pathways; equity considerations refer to inclusion or discussion of older, rural or low-socioeconomic-status participants.
